# PyFgsea: a Rust-powered, fgseaMultilevel-aligned GSEA framework with rolling-window enrichment along single-cell trajectories

**DOI:** 10.1093/bioinformatics/btag257

**Published:** 2026-05-07

**Authors:** Kuanghao Wang, Hong Shi

**Affiliations:** Institute of Primate Translational Medicine, Kunming University of Science and Technology, Kunming 650500, China; Institute of Primate Translational Medicine, Kunming University of Science and Technology, Kunming 650500, China

## Abstract

**Summary:**

GSEA is a standard approach for pathway interpretation, yet Python ecosystems lack a high-performance implementation aligned with the fgseaMultilevel rare-event estimator target, especially for trajectory-aware rolling-window analysis. Under matched inputs, PyFgsea remains near-identical for normalized enrichment scores (NES; Pearson r>0.999), machine-precision identical for enrichment scores (ES), and statistically faithful for nominal *P* values relative to the R fgseaMultilevel reference. Its stateful rolling-window engine further reduces repeated trajectory-analysis overhead, yielding ∼1.9-fold end-to-end wall-time speedup in a conservative stress test and, in a narrower 100-window component benchmark, up to 7.47-fold acceleration. Rolling-window significance is controlled only by within-window Benjamini–Hochberg correction across pathways rather than by trajectory-wide global error control, so these profiles are intended primarily for local trend exploration and candidate-pathway prioritization.

**Availability and implementation:**

Source code is available at https://github.com/shayuanxukuang/pyfgsea and via PyPI (pip install pyfgsea). An archival snapshot of the code and benchmark data is available on Zenodo (DOI: 10.5281/zenodo.19446446).

## 1 Introduction

Gene set enrichment analysis (GSEA) is widely used to interpret genome-wide expression profiles by quantifying whether a gene set is enriched toward the top or bottom of a ranked list (Subramanian et al. 2005). In Python, commonly used implementations such as GSEApy (Fang et al. 2023) and BlitzGSEA (Lachmann et al. 2022) offer accessible workflows but may differ in runtime, memory, or the behavior of extreme-tail *P* values under multilevel sampling.

The fgsea R package provides an efficient multilevel adaptive sampling procedure (“fgseaMultilevel”) that targets accurate rare-event *P* values without requiring extremely large numbers of permutations (Korotkevich et al. 2021). However, a statistically aligned, high-performance implementation has been missing from the Python ecosystem. This gap becomes more pronounced in single-cell trajectory analysis, where enrichment is often desired along pseudotime using rolling windows, resulting in hundreds to thousands of GSEA evaluations.

**Figure 1:**
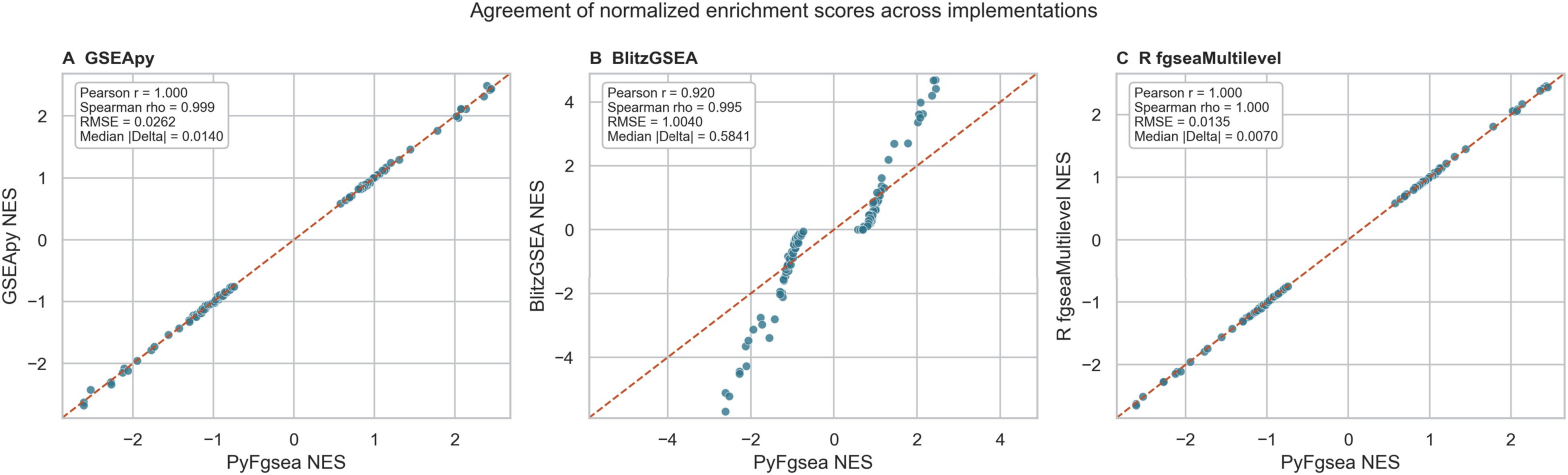
Agreement of normalized enrichment scores (NES) across implementations on matched synthetic benchmark inputs. Panels compare PyFgsea with (A) GSEApy, (B) BlitzGSEA, and (C) R fgseaMultilevel. Dashed lines indicate identity; insets report concordance statistics. PyFgsea remained near-identical for NES relative to the R reference.

**Figure 2:**
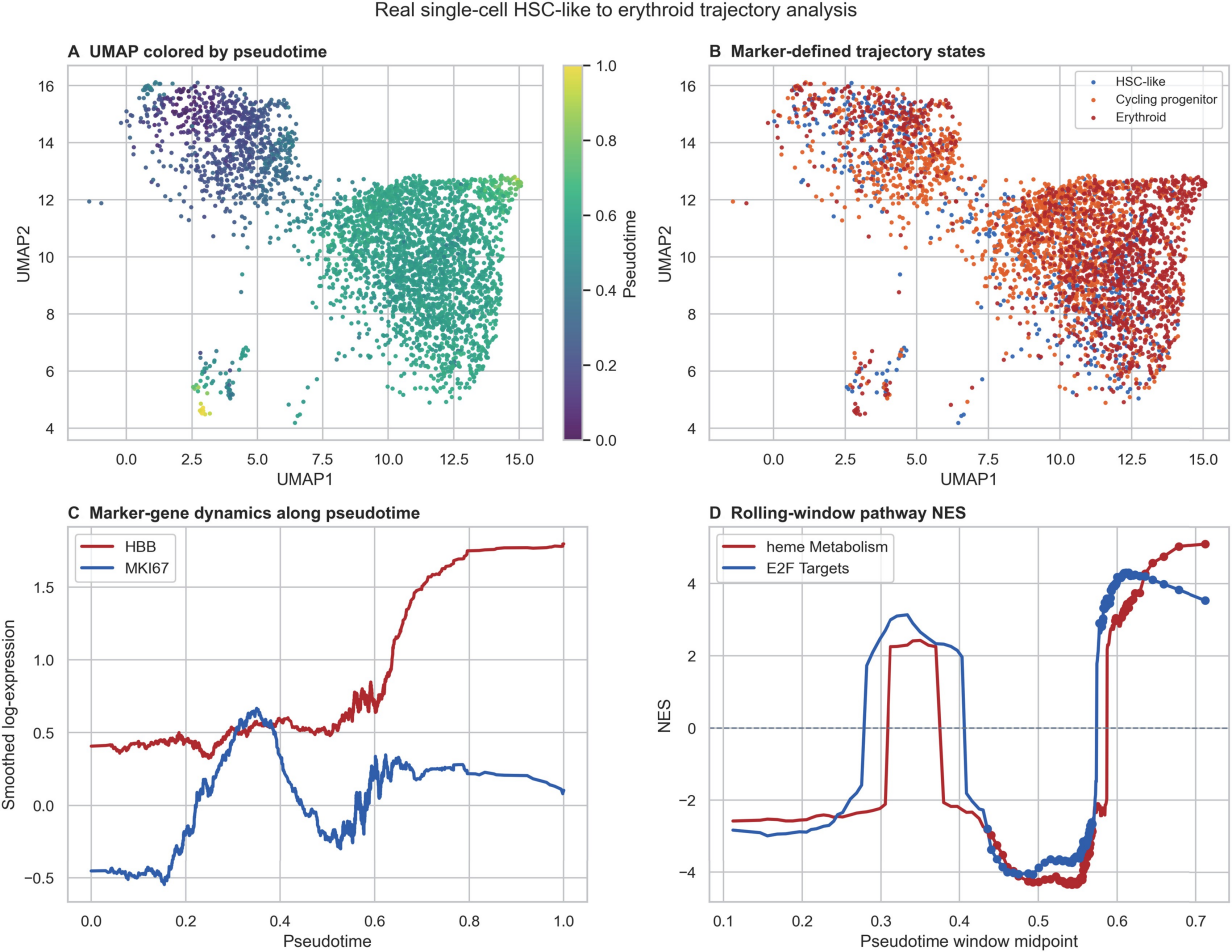
Rolling-window GSEA along a GSE155254-derived HSC-like-to-erythroid trajectory. Cells were ordered by diffusion pseudotime (DPT) and analyzed with PyFgsea using the default full-window workflow. (A) UMAP colored by pseudotime. (B) Marker-defined cell states. (C) Smoothed expression of representative genes HBB and MKI67 along pseudotime. (D) Window-wise NES for representative pathways (heme metabolism and E2F targets) across the ordered trajectory; filled points indicate within-window FDR <0.05 after Benjamini–Hochberg correction across pathways. Terminal-truncation behavior is audited separately in Fig. S14, available as supplementary data at *Bioinformatics* online.

PyFgsea is a high-performance framework built on a Rust backend with PyO3 bindings. Its main contribution is practical rather than methodological: it provides (i) a Python-facing implementation aligned with the fgseaMultilevel rare-event estimator target, (ii) a multi-threaded architecture that substantially reduces runtime and memory footprint, and (iii) a specialized stateful rolling-window runner that minimizes redundant computations for efficient trajectory analysis. The cross-implementation concordance analysis and the rolling-window trajectory case study are summarized in Figs 1 and 2, respectively.

## 2 Materials and methods

### 2.1 Core multilevel workflow

PyFgsea re-implements the fgseaMultilevel rare-event estimator target with the same ES definition, null-pathway construction, and multilevel tail factorization (Korotkevich et al. 2021); in the current Rust release, the conditional levels are realized through empirical median thresholding and elite-clone rejuvenation. A complete methodological workflow diagram is provided in Fig. S7, available as supplementary data at *Bioinformatics* online.

For a pathway *S* of size *K* in a ranked list of *N* genes with ranked statistics $\left\{r_{j}\right\}$, PyFgsea uses the same weighted preranked enrichment score as in classical GSEA and fgseaMultilevel (Subramanian et al. 2005, Korotkevich et al. 2021):


$$\begin{array}{c} ES=\underset{1\leq i\leq N}{\max}\left(P_{\text{hit}}(i)-P_{\text{miss}}(i)\right),\\ P_{\text{hit}}(i)=\frac{\sum_{\begin{array}{c} j\leq i\\ j\in S \end{array}}|r_{j}{|}^{p}}{\sum_{g\in S}|r_{g}{|}^{p}},\\ P_{\text{miss}}(i)=\frac{\sum_{\begin{array}{c} j\leq i\\ j\notin S \end{array}}1}{N-K}. \end{array}$$

Observed and null pathways are compared under the same sign convention as the observed walk, so positive and negative enrichments are handled symmetrically. Null pathways are generated by uniformly sampling *K* genes without replacement from the valid ranked-gene universe. The rare-event tail probability is then estimated through the same multilevel factorization (Korotkevich et al. 2021),


$$P(ES\geq ES_{\text{obs}})\approx P_{0}\prod_{l=1}^{L}P(ES\geq c_{l}|ES\geq c_{l-1}).$$

Here, $P_{0}$ denotes the baseline tail probability from the initial samples, and $c_{l}$ denotes progressively stricter conditional ES thresholds. If the observed pathway is not yet in the rare tail, the direct baseline estimate is returned instead of further splitting. The current release therefore combines a baseline sample, empirical median-conditioned levels, elite-clone rejuvenation, and budget/precision/floor-based stopping. The Python API adheres to standard GSEA conventions: users provide ranked genes, a GMT gene set collection, and parameters controlling multilevel estimation (e.g. eps, sample_size, seed). PyFgsea differs primarily at the implementation layer (Rust/PyO3 backend, Rayon scheduling, and deterministic pathway-local PRNG streams) and in the functional extension of a stateful rolling-window runner for trajectory analysis; Table S5 and Section S8, available as supplementary data at *Bioinformatics* online provide a concise same-versus-different summary and fuller algorithmic details.

### 2.2 Rust backend and parallelism

The computational core is implemented in Rust and exposed to Python via PyO3. Pathway-level evaluations are parallelized across threads using a dynamic work-stealing strategy. To guarantee deterministic reproducibility across thread counts under matched inputs and a fixed master seed without synchronization blocking, PyFgsea derives independent, deterministic pseudo-random number generator (PRNG) streams for each pathway by hashing a user-provided global master seed with the unique pathway identifier (see Section S9, available as supplementary data at *Bioinformatics* online). Memory allocations are minimized by reusing buffers across pathways and windows. This design targets both speed and reduced peak RAM for large gene-set collections.

### 2.3 Rolling-window GSEA for single-cell trajectories

For trajectory analysis, PyFgsea supports rolling-window enrichment along pseudotime. Cells are ordered by pseudotime (e.g. diffusion pseudotime, DPT (Haghverdi et al. 2016)) computed using Scanpy (Wolf et al. 2018). A window of fixed size slides along the ordered cells with a user-defined step size. Trajectory termini are handled without padding, and Fig. S14, available as supplementary data at *Bioinformatics* online additionally audits an explicit terminal-truncation extension. The choice of window and step size governs temporal resolution and statistical stability. As a practical starting range that should be tuned to trajectory length and noise level, an initial window size spanning 5%–10% of the total cells and a step size of 1%–2% of total cells are suggested. Smaller windows may be overly sensitive to single-cell noise, while excessively large windows risk oversmoothing biologically transient dynamics (a comprehensive parameter sensitivity analysis is provided in Fig. S8 and Sections S10 and S13, available as supplementary data at *Bioinformatics* online). The erythroid illustration in Fig. 2 intentionally uses a somewhat larger 500-cell baseline window (∼14% of that analyzed trajectory) because this lineage segment is relatively short and noisy, and the figure prioritizes a smoother descriptive profile over maximal temporal resolution. For each sliding window along the pseudotime-ordered cells, genes are ranked by a logFC-like score computed on log1p-normalized expression: $S_{g}=\mu_{g,in}-\mu_{g,\mathrm{out}}$, where $\mu_{g,in}$ and $\mu_{g,\mathrm{out}}$ denote the mean expression of gene *g* inside the current window and in all remaining cells, respectively. The ranked list is then used as input to preranked GSEA over a chosen gene set collection (e.g. MSigDB Hallmark (Liberzon et al. 2011, 2015)). Within each window, Benjamini–Hochberg adjustment is applied across pathways, so window-level significance corresponds to within-window FDR control rather than trajectory-wide error control across repeated windows. Rolling-window findings should therefore be interpreted as exploratory/descriptive summaries unless supported by orthogonal evidence. The rolling-window workflow is implemented in pyfgsea.trajectory.run_trajectory_gsea, and the repository includes a demonstrative script at examples/trajectory_demo.py (release tag: v0.1.4).

### 2.4 Datasets and benchmarking

Performance and faithfulness were evaluated on both bulk-like and single-cell settings. For the single-cell case study shown in Fig. 2, a processed HSC-like-to-erythroid trajectory derived from public accession GSE155254 was used. Benchmarking compared PyFgsea with GSEApy (Fang et al. 2023), BlitzGSEA (Lachmann et al. 2022), and the reference R fgsea implementation (Korotkevich et al. 2021) under matched inputs and parameters (see Material, available as supplementary data at *Bioinformatics* online for detailed parameter alignment).

Benchmarks were conducted on a server equipped with dual Intel Xeon Platinum 8352Y CPUs (64 cores/128 threads, @2.20 GHz) and 1.5 TiB RAM, running Ubuntu 22.04.1 LTS. Software versions were Python 3.10.19, R 4.4.3 (fgsea 1.32.2), GSEApy 1.1.11, and BlitzGSEA 1.3.54. To ensure fairness, multithreading was controlled: PyFgsea and Python baselines used 4 threads (via RAYON_NUM_THREADS = 4 or OMP_NUM_THREADS = 4), while R/fgsea was configured with nproc = 4 (using BiocParallel). Each condition was measured in three independent repeats using independent processes to minimize cache interference. For the cross-tool benchmarks summarized in Table S4, available as supplementary data at *Bioinformatics* online, runtime was measured as wall-clock elapsed time around each benchmark invocation, and peak memory was estimated by repeated polling of the benchmark process RSS (including child processes) during execution; means over three repeats are reported in Table S4, available as supplementary data at *Bioinformatics* online. Detailed performance breakdowns, including thread scaling and rolling-window component analysis, are provided in the Material, available as supplementary data at *Bioinformatics* online.

## 3 Results

### 3.1 Performance and memory footprint

Across representative dataset sizes (spanning small, medium, and large scales), PyFgsea consistently reduced runtime while using substantially less memory than alternative Python implementations (Table S4, available as supplementary data at *Bioinformatics* online). In the large benchmark (20k genes, 5k gene sets), PyFgsea completed in 1.35 s with 68.0 MB PeakRSS, compared with 18.00 s and 611.4 MB for BlitzGSEA and 189.42 s and 5891.1 MB for GSEApy. These gains are primarily attributable to the Rust backend, pathway-level parallelism, and careful reuse of intermediate buffers.

### 3.2 Statistical faithfulness to fgseaMultilevel

To assess equivalence to the reference implementation, enrichment scores (ES) and normalized enrichment scores (NES) were compared against R fgseaMultilevel (Korotkevich et al. 2021). Across matched inputs, PyFgsea remained near-identical for NES, machine-precision identical for ES, and statistically faithful for transformed nominal *P* values relative to the R reference (Fig. 1). NES concordance remained uniformly very high (Pearson r=1.000; Spearman ρ=1.000; RMSE =0.0135; median absolute difference |Δ|=0.0070; Fig. 1C). ES values were numerically identical within machine precision. In the synthetic benchmark summary shown in Fig. 1, transformed nominal *P* values remained statistically faithful to the reference implementation (overall Pearson r=0.997; Spearman ρ=0.988; RMSE =0.1696; median |Δ|=0.0467 on $-{\;\text{log}\;}_{10}(p)$), although sparse local trajectory windows could show visibly larger dispersion than ES or NES (worst-window Pearson r=0.941 on $-{\;\text{log}\;}_{10}(p)$; Table S7, available as supplementary data at *Bioinformatics* online). In such sparse local windows, ES and NES should therefore be regarded as the primary cross-implementation comparanda, whereas agreement in transformed nominal *P* values is more sensitive to tail-estimation noise and pathway-count filtering. Despite this, downstream pathway calls remained stable overall across validation regimes, with top-10 |*NES*| pathway overlap of 0.90–1.00 and BH-FDR < 0.05 pathway-set overlap of 0.86–1.00 between PyFgsea and the R reference (Table S7, available as supplementary data at *Bioinformatics* online). For multilevel *P* values, PyFgsea employs a stricter default floor (eps = 1e-50) compared to R’s default (eps = 1e-10), allowing for higher resolution in extreme tails. When eps is matched, differences in deep-tail *P* values are generally modest, but they become more apparent when few pathways pass size filters or when strongly enriched local windows occupy sensitive rare-event tails (Figs S3 and S4, available as supplementary data at *Bioinformatics* online). Under matched inputs, PyFgsea, therefore, preserved near-identical NES, machine-precision ES agreement, and statistically faithful nominal *P* values while additionally offering extended precision for rare events.

### 3.3 Trajectory case study: rolling-window enrichment

PyFgsea enables rolling-window enrichment along pseudotime for large single-cell datasets. In a real GSE155254-derived HSC-like-to-erythroid trajectory, rolling-window GSEA provided an illustrative view of temporally localized pathway-profile variation while maintaining feasible runtimes over hundreds to thousands of windows (Fig. 2). Across rolling-window benchmarks, the stateful runner yielded ∼1.9-fold speedup in a conservative end-to-end stress test and up to a 7.47-fold acceleration in a narrower 100-window component benchmark. Figure 2 uses the default full-window workflow without the optional terminal-truncation extension, which is evaluated separately in Fig. S14, available as supplementary data at *Bioinformatics* online. Because significance in such plots is controlled only within each window across pathways rather than across the full trajectory, these rolling-window profiles should be interpreted primarily as a local exploratory screen for trend visualization and candidate-pathway selection rather than as trajectory-wide significance discovery.

## 4 Discussion

PyFgsea brings an fgseaMultilevel-aligned implementation to the Python ecosystem and extends it to rolling-window trajectory analysis. When interpreting trajectory dynamics, users should be mindful of the inherent limitations of the rolling-window ranking statistic. By summarizing local expression changes as a simple difference of means, it inherently assumes monotonic expression shifts within the evaluated window. Consequently, genes exhibiting complex, non-monotonic dynamics outside the local window may have their relative changes underestimated, potentially causing false-negative enrichments. Conversely, sparse genes with high technical variance may dominate local rankings, risking false positives. Dynamic pathways should therefore be interpreted cautiously, cross-referenced with smoothed single-gene visualizations, and treated as exploratory/descriptive unless supported by orthogonal evidence. In addition, fixed cell-count windows do not correspond to a fixed biological time span when cell density varies across pseudotime.

Future work includes broader input conventions, additional trajectory ranking statistics, adaptive or variable-width windows for non-uniform pseudotime sampling, and more diverse cross-platform validation.

## Supplementary Material

btag257_Supplementary_Data

## Data Availability

All scripts and minimal example data required to reproduce the reported software benchmarks and rolling-window workflow are provided in the public PyFgsea repository under repro/, examples/, and the package source tree (see *Availability and implementation*). Public reference resources used in the showcase analyses include the GSE155254-derived erythroid trajectory example and MSigDB hallmark gene sets; access and licensing follow the respective providers’ terms.

## Availability and implementation

PyFgsea is released under the MIT License. Source code, documentation, and tutorials are available at https://github.com/shayuanxukuang/pyfgsea. The Python package can be installed directly from PyPI (pip install pyfgsea) and supports Linux, Windows, and macOS. A permanent archival snapshot is also available at Zenodo: 10.5281/zenodo.19446446.
