# Supplementary material for: PyFgsea: a Rust-powered, fgseaMultilevel-aligned GSEA framework with rolling-window enrichment along single-cell trajectories
Source: Bioinformatics. 2026 May 7;42(5):btag257. doi: 10.1093/bioinformatics/btag257 (PMC13197117; doi:10.1093/bioinformatics/btag257)
Supplement: btag257_Supplementary_Data [file btag257_supplementary_data.pdf]

Supplementary Information for "PyFgsea: A Rust-Powered, `fgseaMultilevel`-Aligned GSEA Framework with Rolling-Window Enrichment Along Single-Cell Trajectories"

Kuanghao Wang Hong Shi

Supplementary Methods

S1 Benchmark protocol

Benchmarks were executed on a single machine with CPU: Intel Xeon Platinum 8352Y @ 2.20GHz (64 physical cores / 128 hardware threads), RAM: 1.5 TiB, OS: Ubuntu 22.04.1 LTS (kernel 6.2.0-34-generic). Software stack: Python 3.10.19 with PyFgsea (this work, local build), R 4.4.3 with fgsea 1.32.2, GSEApy 1.1.11, BlitzGSEA 1.3.54, and common scientific Python dependencies (NumPy/Pandas). Thread counts were standardized per tool: PyFgsea used the Rayon pool via `RAYON_NUM_THREADS`; Python baselines controlled BLAS/OpenMP threads via `OMP_NUM_THREADS` and `MKL_NUM_THREADS`; R/fgseaused4threads. For each condition (dataset scale × tool), we performed 3 independent repeti-tions; the main manuscript re ports means, and mean ± SD are given in Supplementary Table S4. Runtime was measured using /usr/bin /time `-v` and/or `time.perf counter`; peak resident set size (PeakRSS) was taken from /usr/bin/time `-v` (Maximum resident set size) and/or periodic sampling at 100 ms reso-lution. Inputs used the sa me pretrained ranked gene lists and the MSigDB Hallmark gene sets; gene universes were matched by intersecting pathways with the ranked list before analysis to ensure end-to-end comparability.

S2 Parameter harmonization

Table S1 summarizes key parameters matched across implementations, including gene-set filters, tie handling, multilevel settings, NES normalization, thread control, and multiple testing.

S3 Single-cell trajectory details

Dataset: hematopoietic differentiation lineage from the Human Cell Atlas; cells were quality-controlled, highly variable genes selected, neighborhood graph constructed, and pseudotime inferred using diffusion pseudotime (DPT) in Scanpy. Root cells were chosen from the stem-like compartment; lineage assignment followed annotation metadata. Rolling-window enrichment used window_size=500 cells and step=50 cells along the pseudotime order; for each window, a preranked gene list was computed with a signed statistic difference-of-meansonlog1p-normalizedexpression(logFC-like). Pathway activity curves report NES a s a function of pseudotime; significant windows were defined by FDR < 0.05. Windows advance by fixed cell counts, so pseudotime density variations can yield nonuniform window coverage; we therefor e retain the full gene ranking per window and keep the gene universe fixed across the trajectory to ensu re reproducibility.

Supplementary Results

S4 Additional agreement analyses

Figure S1 compares subset-level agreement between PyFgsea and the R reference for representative groups in HCA and PBMC. Each subset reports correlations and absolute error summaries across pathways, ensuring that global aggregation does not mask group-specific deviations. Subset-level analyses confirm that ES/NES alignment holds across diverse partitions and that transformed nominal P-values show strong concordance with moderate dispersion confined to the extreme tail.

Table S1: Key parameters harmonized across implementations in comparative benchmarks (noted exceptions).

Aspect	PyFgsea	R fgsea	GSEApy
Gene-set size	min_size=15, max_size=500	minSize=15, maxSize=500	matched
Ranking ties	random order (seeded)	random order (seeded)	default
Multilevel settings	sample_size=101, eps=10^{-50}, seed=42	sampleSize=101, eps=10^{-10}, seed=42	n/a
NES normalization	nperm_nes=1000	permutations=1000	nperms=1000
Thread control	RAYON_NUM_THREADS	BiocParallel nproc=4	OMP/MKL env
Multiple testing	BH (FDR)		BH (FDR)

Unless otherwise noted, Table S1 summarizes the harmonized settings used in the main benchmark suite. For the deep-tail nominal-P audits in Figures S2-S4, an analysis-specific lower nominal-P floor was used (as indicated in the relevant figure title) to avoid truncation of transformed tail values; this override is distinct from the default eps settings summarized here.

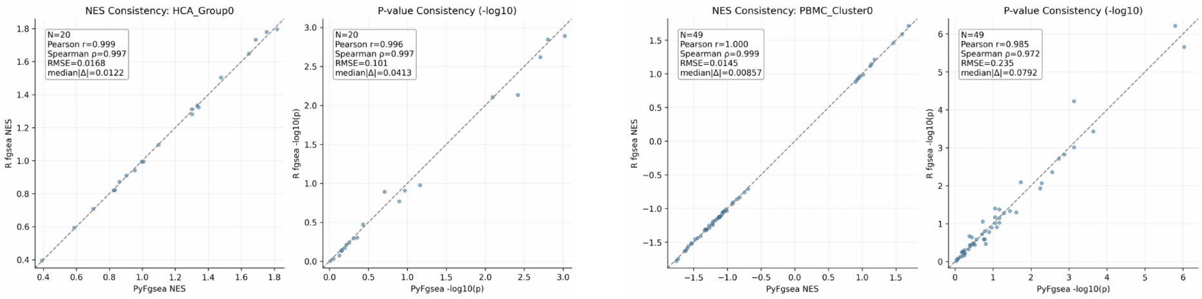

Figure S1: Subset-level agreement between PyFgsea and R `fgsea`. Left: HCA_Group0. Right: PBMC_Cluster0.

S5 Extreme-tail / rare-event stress tests

Figure S3 shows deep-tail consistency under Monte Carlo seed variability: PyFgsea's $-\log_{10}(P)$ falls within the R inter-seed empirical 95% interval for the vast majority of highly significant pathways, indicating behavior consistent with intrinsic seed-to-seed variability. Figure S4 examines budget sensitivity on representative outliers; increasing multilevel `sample_size` changes $\Delta(-\log_{10} P)$ only modestly, reflecting combined effects from RNG streams, stopping criteria, tie handling, and subtle preprocessing intersections in multilevel tail estimation.

S6 Calibration and stability

Figure S5 shows that both execution modes remained close to the null expectation, with KS = 0.013 for the standard mode and KS = 0.030 for the batched mode. The batched mode therefore showed only a modest increase in null-calibration deviation relative to the standard mode, with departures concentrated primarily in the deepest tail. Figure S6 summarizes run-to-run stability across repeated executions; transformed nominal P-values show tight distributions across replicates, indicating low variance in typical regimes.

Thread scaling on the Large setting shows substantial runtime reduction up to 8 threads, followed by saturation due to parallel overheads and memory bandwidth limits; PeakRSS increases only modestly, indicating that multithreading does not induce disproportionate memory growth. For rolling-window throughput, we report a decomposed benchmark that separates preprocessing, NES background construction, and the multilevel core; the stateful runner amortizes window-invariant components and yields an end-to-end speedup aligned with the main manuscript.

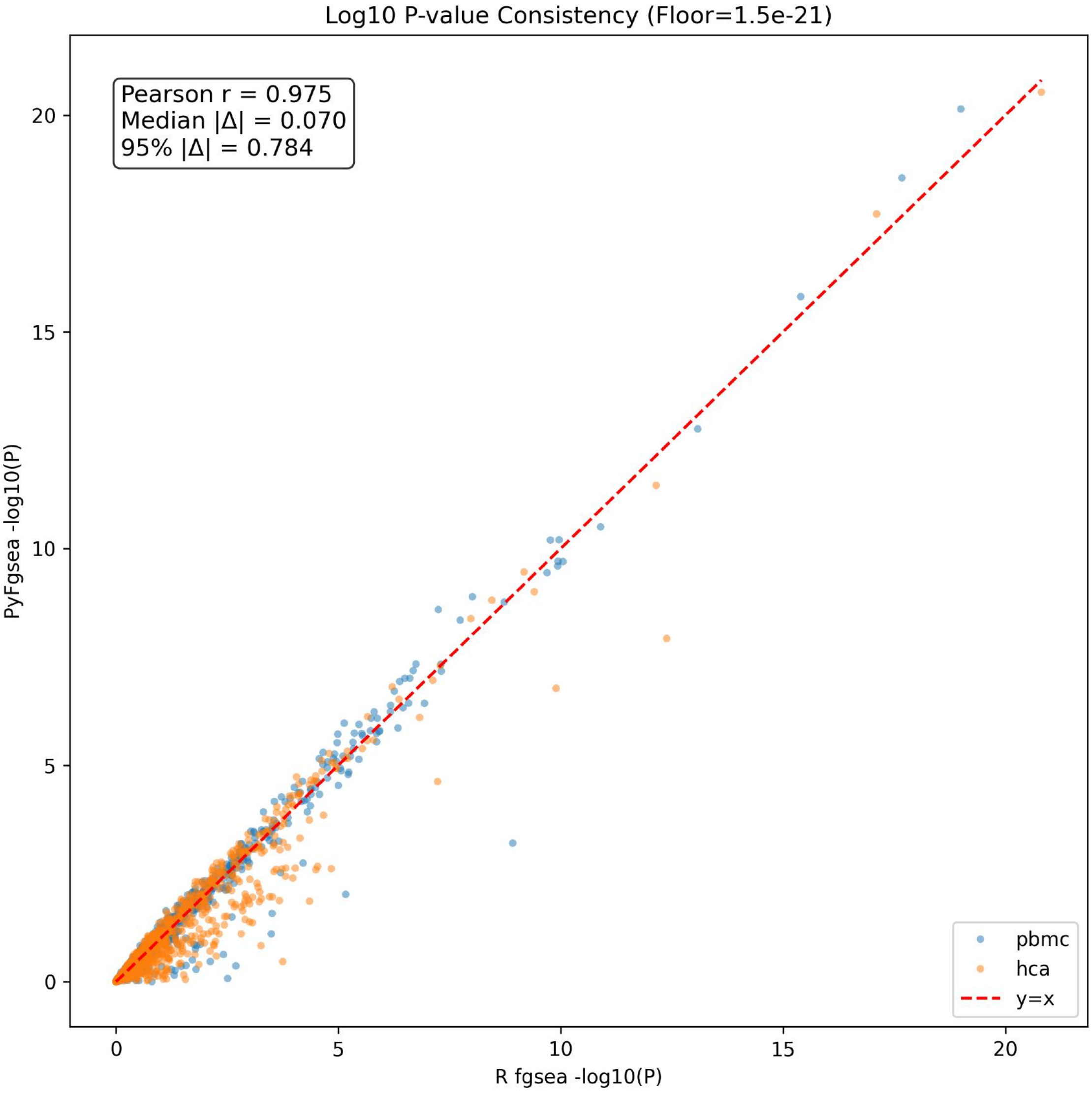

Figure S2: Extended $-\log_{10}(P)$ consistency across subsets and pathways.

Note: This deep-tail nominal-P audit uses an analysis-specific lower floor for transformed tail values and is therefore not directly tied to the default eps entry listed in Table S1.

Table S2: Thread scaling of PyFgsea on the Large setting (20k genes, 5k gene sets).

Threads	Runtime (s)	PeakRSS (MB)
1	11.49	54.7
2	6.02	55.2
4	3.72	55.6
8	2.52	56.3
16	2.33	58.0

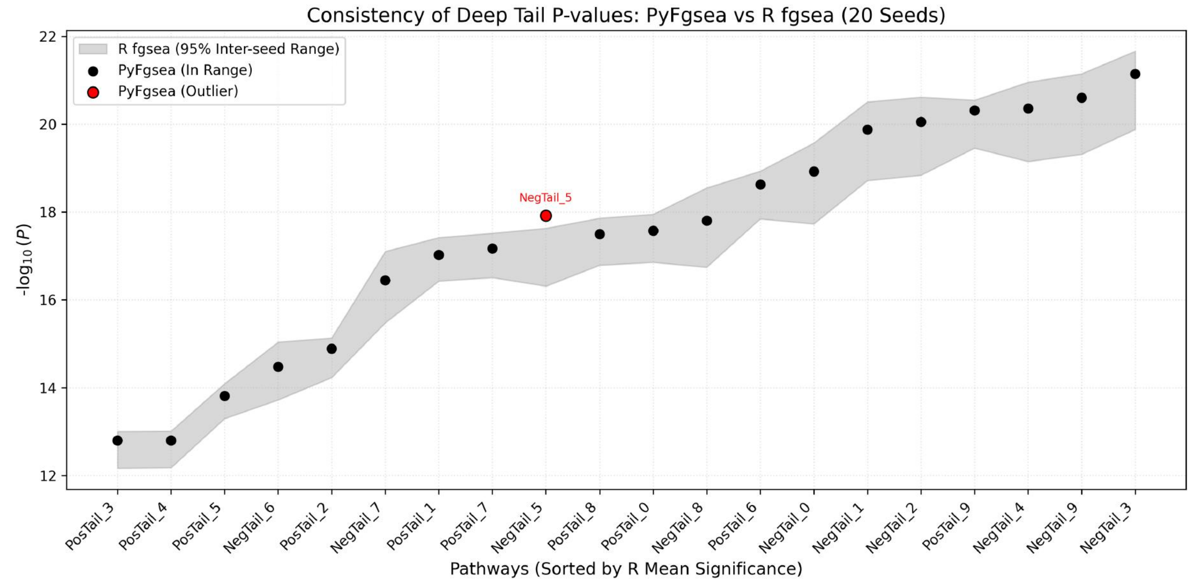

Figure S3: Deep-tail multilevel P-value consistency under Monte Carlo seed variability (rare-event setting).

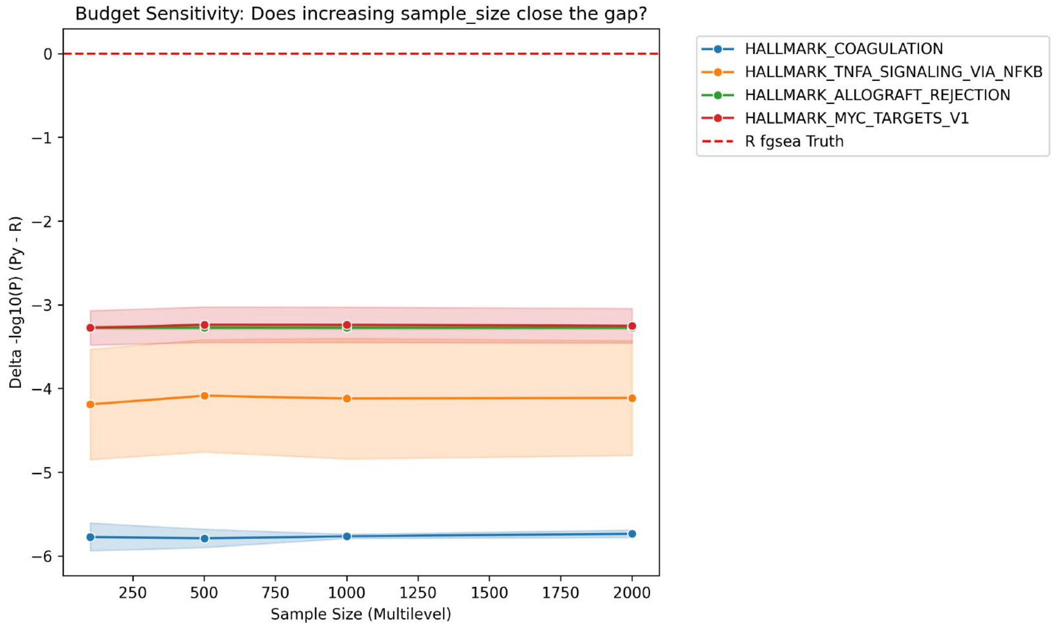

Figure S4: Budget sensitivity analysis for representative tail outliers under varying multilevel sampling settings.

Table S3: End-to-end rolling-window throughput benchmark (100 windows, 3 repeats, 5000 pathways, 20,000 genes). Standard denotes stateless per-window re-initialization; Optimized reuses a stateful runner across windows to amortize window-invariant costs.

Metric	Standard (Stateless)	Optimized (Stateful)
Preprocessing (s)	4.122 ± 0.022	0.044 ± 0.001
NES background (s)	46.604 ± 0.117	0.047 ± 0.010
Multilevel core (s)	11.192 ± 0.052	8.080 ± 0.068
Total time (s)	62.288 ± 0.119	8.335 ± 0.089

Overall speedup: 7.47× (62.288 / 8.335). Consistency note: The decomposed timings align with the main manuscript (Results—Performance overview) for cross-check during review.

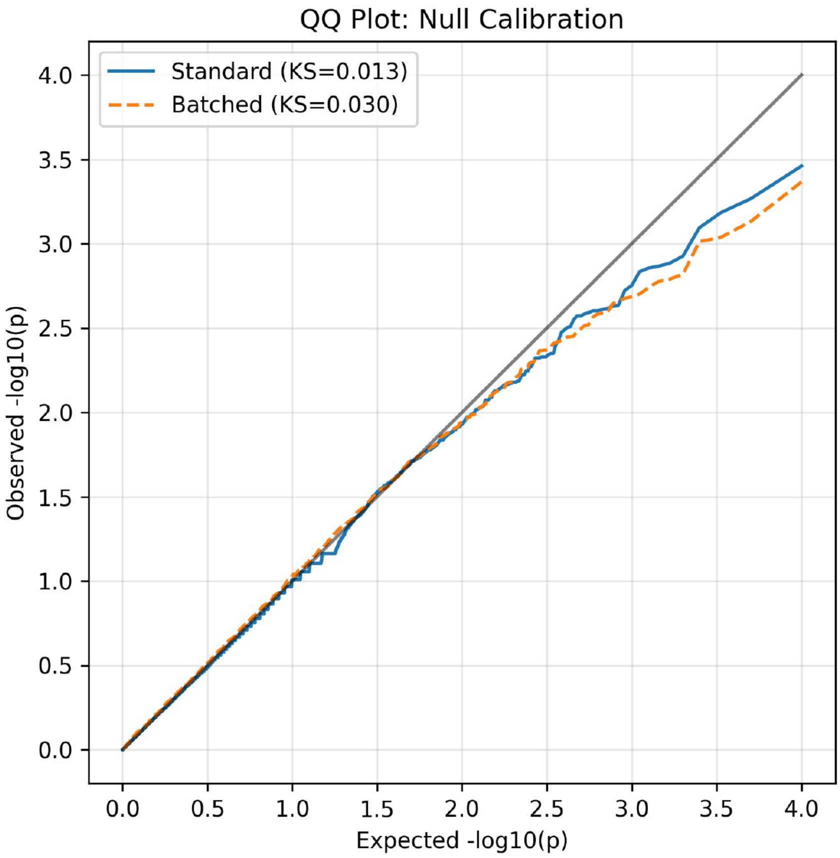

Figure S5: Null calibration (QQ plot) for nominal P-values under alternative execution modes.

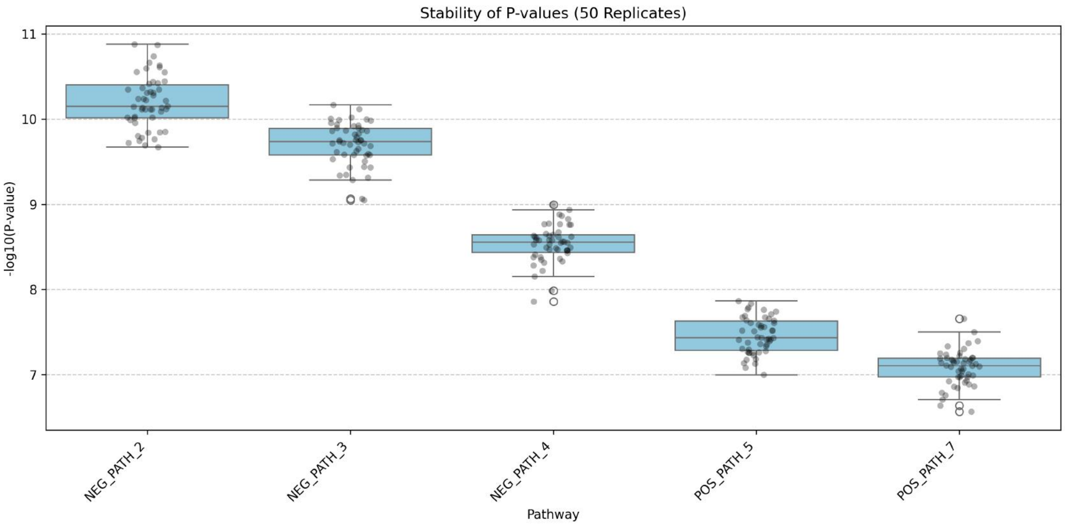

Figure S6: Run-to-run stability across repeated executions (distribution of $-\log_{10}(P)$ deviations).

Table S4: Runtime and peak memory usage comparison across implementations (example settings). All tools (including R/fgsea) were run with 4 threads.

Dataset	Metric	PyFgsea	GSEApy	BlitzGSEA	R fgsea
Small (12k genes, 200 sets)	Time (s)	0.16	5.06	5.22	6.37
Small (12k genes, 200 sets)	RAM (MB)	49.9	270.0	570.0	1309.3
Medium (20k genes, 1k sets)	Time (s)	0.35	22.57	7.06	5.62
Medium (20k genes, 1k sets)	RAM (MB)	53.7	1282.6	583.5	1414.5
Large (20k genes, 5k sets)	Time (s)	1.35	189.42	18.00	8.42
Large (20k genes, 5k sets)	RAM (MB)	68.0	5891.1	611.4	1620.0

S8. Core Mathematical Formulations and Multilevel Adaptive Sampling

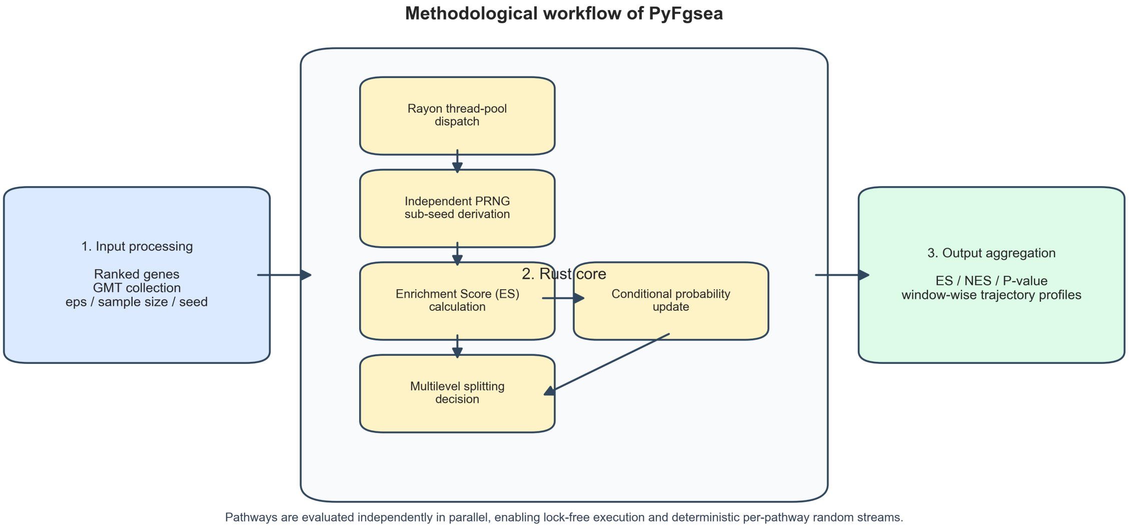

Figure S7: Methodological workflow diagram. PyFgsea accepts ranked genes, a GMT collection, and multilevel estimation parameters as input; executes pathway-wise tasks in the Rust core using Rayon scheduling, deterministic per-pathway PRNG sub-seeds, Enrichment Score (ES) evaluation, and multilevel splitting logic; and finally aggregates ES/NES/P-value outputs across pathways and rolling windows.

Enrichment Score (ES) Calculation. For a ranked list of N genes and a gene set S of size K, the ES is defined as the maximum deviation from zero of a weighted random walk:

$$ES = \max_{1 \leq i \leq N} \left(P_{\text{hit}}(i) - P_{\text{miss}}(i) \right).$$

Using the standard weighted preranked GSEA notation, with ranked statistics $\{r_j\}_{j=1}^{N}$ and weight exponent p,

$$P_{\text{hit}}(i) = \sum_{\substack{j \leq i \\ j \in S}} \frac{|r_j|^p}{\sum_{g \in S} |r_g|^p}, \qquad P_{\text{miss}}(i) = \sum_{\substack{j \leq i \\ j \notin S}} \frac{1}{N - K}.$$

Thus, $P_{\text{hit}}(i)$ accumulates the normalized weighted contribution of genes in the pathway up to rank i, whereas $P_{\text{miss}}(i)$ accumulates the complementary background walk over genes outside the pathway.

Random Pathway Generation. To build the background null distribution, PyFgsea generates random pathways by uniformly sampling K genes without replacement from the given gene universe (all valid genes present in the input ranked list).

Multilevel Adaptive Sampling. The algorithm efficiently targets rare-event P-values by iterative splitting.

Initialization. An initial set of independent permutations (N_{base}, e.g. 1000) is evaluated to form a baseline null distribution.

Iterative Splitting Rules. If the observed ES falls in the extreme tail, i.e. the estimated P-value approaches the resolution limit of the current sample size, the state space is split. Random walks that reach the current top threshold are cloned, and their remaining trajectories are simulated to conditionally explore rarer regions.

P-value Formula. The final tail probability is computed as the product of conditional probabilities across all L splitting levels:

$$P \approx P_0 \prod_{l=1}^{L} P(ES \geq c_l \mid ES \geq c_{l-1}) \,.$$

Stopping Conditions. Sampling recursively stops when the relative error (standard deviation) of the estimated P-value converges below a user-defined threshold, the computational budget is exhausted, or the calculated P-value drops below the absolute floor `eps` (e.g. 10^{-50}).

Algorithm S1. Pseudocode-style summary of the PyFgsea multilevel routine. The following boxed summary is intended to make the current Rust implementation more directly auditable. It describes the release-`v0.1.4` multilevel workflow at a pseudocode level, while Table S5 separately clarifies which components are conceptually preserved from `fgseaMultilevel` and which are implementation-specific.

Inputs: ranked scores $\{r_j\}_{j=1}^N$, observed pathway hits S with size K, observed metric m_{obs} derived from ES_{obs}, baseline sample size N_{base}, master seed, `eps`.

Initialization:

1. Draw N_{base} random size-matched pathways uniformly without replacement from the valid gene universe.
2. Compute each null-pathway ES and its tail metric m_i using the same sign convention as the observed pathway.
3. Count $b_0 = \#\{i : m_i \geq m_{\text{obs}}\}$. If $b_0 \geq \lceil 0.05 N_{\text{base}} \rceil$, return the direct estimate $(b_0+1)/(N_{\text{base}}+1)$.

Iterative multilevel splitting:

1. At level l, set the current threshold c_l to the empirical median of the population metric values, i.e. the cutoff at index $N_{\text{base}}/2$ after partial sorting.
2. Let $b_l = \#\{i : m_i \geq m_{\text{obs}}\}$. If $c_l \geq m_{\text{obs}}$ or $b_l = N_{\text{base}}$, stop and return $\exp(\log P_{l-1}) \times b_l/N_{\text{base}}$.
3. Update the accumulated log-probability by the elite-survival factor, $\log P_l \leftarrow \log P_{l-1} + \log\left(\frac{N_{\text{base}}/2}{N_{\text{base}}}\right)$.
4. Retain the top half of the current population as elites.
5. For each non-elite slot, clone one elite pathway and rejuvenate it by a short constrained random walk of $w = \text{clamp}(K, 10, 100)$ swap attempts:
   (a) remove one in-pathway gene and propose one out-of-pathway replacement;
   (b) recompute the candidate ES metric;
   (c) accept the proposal only if the candidate metric remains at least c_l; otherwise revert the swap.
6. Record the acceptance rate for that level and continue to the next level until one of the stopping rules is met.

Finalization: apply the absolute floor `eps`, return the estimated nominal P-value, and optionally expose level thresholds and acceptance rates for debugging.

S9. Parallel Architecture and PRNG Management

Task Allocation and Synchronization. Multi-threading is implemented via the Rust `rayon` crate utilizing a dynamic work-stealing thread pool. Parallelization is applied at the pathway level. Since evaluations for different pathways are conditionally independent given the pre-sorted ranked gene list, PyFgsea avoids complex cross-thread synchronization, data-merging locks, or mutexes, resulting in highly scalable execution.

Parallel Random Number Management. A critical challenge in parallel GSEA is ensuring exact statistical reproducibility. PyFgsea avoids a global shared random-state architecture. Instead, the user-provided master seed and the unique pathway string identifier (or index) are hashed together to generate a deterministic, independent sub-seed for each individual pathway. This sub-seed initializes a fast, thread-local PRNG stream. Consequently, under matched inputs and a fixed master seed, the stochastic simulation stream for any specific pathway remains strictly identical across runs despite changes in thread count or OS-level scheduling unpredictability.

Table S5: Same-versus-different summary relative to the original fgseaMultilevel formulation.

Component	Preserved from fgseaMultilevel	Implementation-level difference in PyFgsea	Functional extension in PyFgsea	Expected impact on outputs
ES definition	Same weighted preranked ES statistic and signed random-walk target	Implemented in Rust/PyO3 rather than the R execution stack	None	ES values remain numerically identical within machine precision under matched inputs
Null-pathway generation	Same size-matched random pathway sampling from the valid ranked-gene universe	Deterministic pathway-local sub-seeds are derived from the master seed and pathway identifier	None	Null sampling stays statistically aligned while becoming thread-count invariant
Multilevel tail factorization	Same product-of-conditional-probabilities rare-event estimator	Tail levels are executed in a compiled backend with lightweight object marshalling	None	Deep-tail behaviour follows the same estimator family while improving throughput
Level construction	Same progressively conditioned rare-event levels	Current Rust release realizes levels through empirical median thresholding plus elite-clone rejuvenation under deterministic pathway-local PRNG streams (Algorithm S1)	None	Makes the implemented level schedule explicit while preserving the same rare-event target
Precision policy	Same multilevel sampling logic and stopping interpretation	PyFgsea exposes a stricter default floor (`eps=1e-50`) than the historical R default (`eps=1e-10`)	None	Outputs match in the common range; PyFgsea can additionally report rarer tails at finer resolution
Parallel execution model	Same pathway-wise conditional independence assumption exploited for acceleration	Rayon dynamic work stealing replaces R-side scheduling and avoids cross-thread locks	None	Output values are preserved while runtime scales with thread count under fixed seeds
Trajectory workflow	Same preranked per-window GSEA logic once a ranked vector is formed	Stateful runner reuses pathway definitions and NES background structures across windows	Rolling-window trajectory analysis for pseudotime-ordered single-cell data	Functional scope extends beyond the original single ranked-list workflow without altering per-window GSEA semantics

S10. Rolling-Window Boundary Handling and Sensitivity

Parameter Sensitivity Analysis (Figure S8). Figure S8 now summarizes both window-size and step-size sensitivity across three representative pathways (heme metabolism, E2F targets, and G2-M checkpoint). Small windows exhibit high sensitivity to localized expression changes but are more vulnerable to single-cell technical noise, causing jagged and statistically unstable NES fluctuations. Conversely, overly large windows behave as low-pass filters, over-smoothing the trajectory and potentially masking rapid biological transitions. Step size mainly controls the density of pathway sampling along pseudotime and the computational footprint: coarse steps preserve broad trends but can skip narrow transient peaks, whereas finer steps provide denser temporal localization at higher computational cost.

Guidelines. A practical starting range is a window size covering roughly 5–10% of total cells with a step size near 1–2% of total cells, but this should be tuned to trajectory length and noise level. Step size linearly dictates the computational footprint and temporal granularity. The illustrative erythroid case study in the main text intentionally uses a slightly larger 500-cell baseline window (approximately 14% of that trajectory) because the analyzed lineage segment is relatively short and noisy, and the figure prioritizes a smoother reference profile over maximal temporal resolution. The current erythroid sensitivity grid brackets rather than directly centers this generic starting range, spanning approximately 2.8%, 14.0%, and 28.0% of cells for the tested windows. At the trajectory end, the default production workflow avoids padding-induced edge artifacts by stopping at the last full window; Section S13 below additionally audits a separate explicit terminal-truncation extension.

Table S6: Compact synthetic-benchmark summary of agreement with the R reference, provided to complement the main-text Figure 1 statistics. $\text{Log}_{10}P$ refers to transformed nominal P-values, i.e. $-\log_{10}(P)$.

Metric	Pearson r	Spearman ρ	RMSE	Median $\lvert\Delta\rvert$
NES (PyFgsea vs R)	1.000	1.000	0.0135	0.0070
ES (PyFgsea vs R)	1.000	1.000	0.0000	0.0000
$\text{Log}_{10}P$ (PyFgsea vs R)	0.997	0.988	0.1696	0.0467

S11. Comprehensive Validation and Reproducibility Assessment of PyFgsea

This section provides an integrated validation of PyFgsea across numerical equivalence, stochastic stability, and reproducibility under repeated and parallel execution. The goal is not to repeat the methodological details already summarized in Sections S8–S10, but to make the implementation claims directly testable through a compact, reviewer-oriented validation package.

S11.1 Validation design and benchmark regimes. To make the revised supplement more self-contained, we validated PyFgsea across both synthetic and trajectory-derived ranked lists. The synthetic regimes span small, medium, and large benchmark sizes. The trajectory-derived regimes reuse the erythroid case study from the main text, but extract representative early, middle, and late ranked windows directly from the pseudotime ordering before running standard preranked GSEA against the same Hallmark collection. Across all regimes, pathway size filters, `sampleSize`, and `eps` were harmonized between PyFgsea and R/`fgseaMultilevel`.

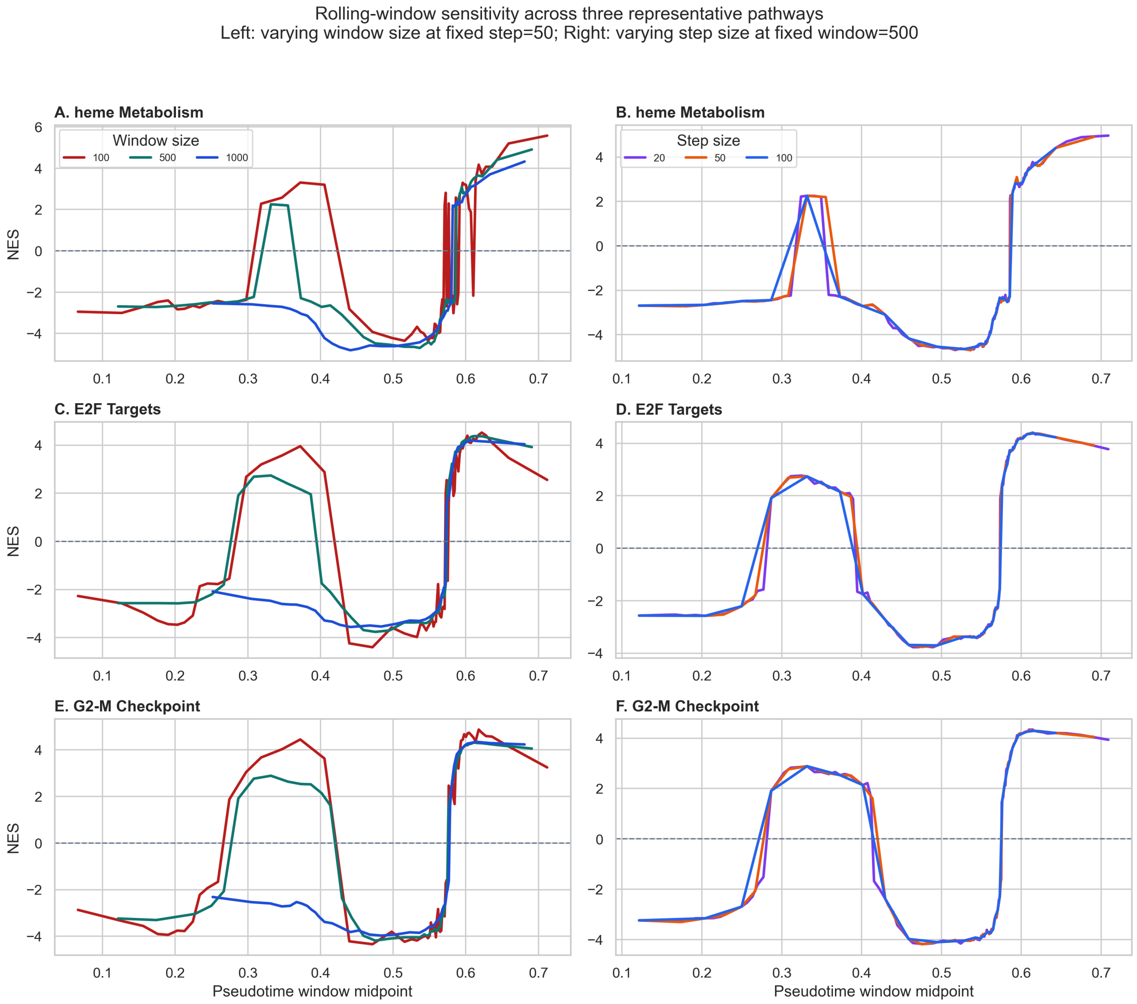

Figure S8: Rolling-window parameter sensitivity across three representative Hallmark pathways in the real erythroid trajectory analysis. Left column: varying window size (100, 500, 1000 cells) at fixed step size 50. Right column: varying step size (20, 50, 100 cells) at fixed window size 500. Smaller windows capture transient local structure but amplify noise, whereas larger windows smooth the curves; coarser step sizes reduce temporal sampling density and can miss short-lived NES peaks.

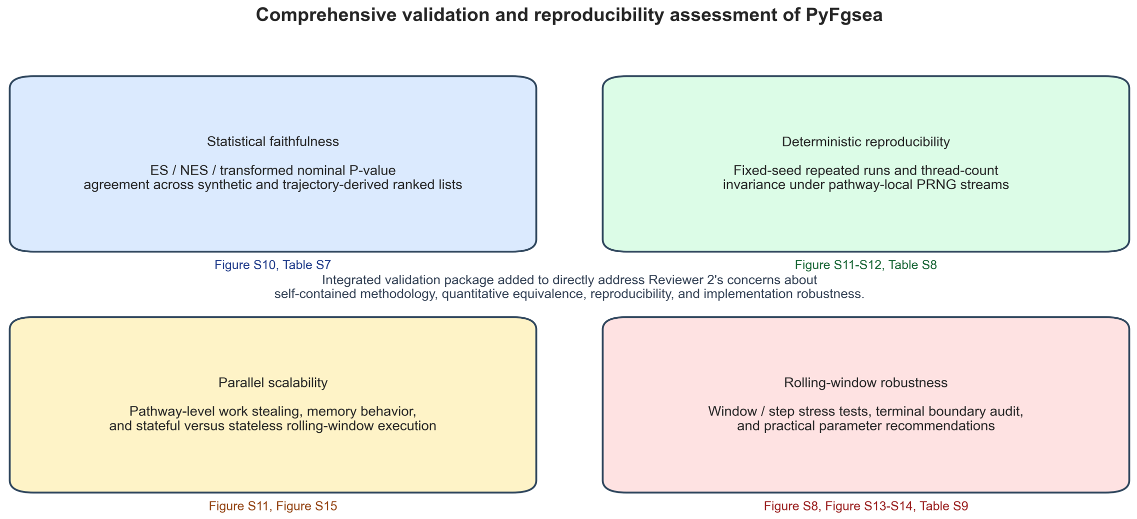

Figure S9: End-to-end validation overview map. The new validation package is organized around four complementary axes: statistical faithfulness to the R `fgseaMultilevel` reference, deterministic reproducibility under repeated and parallel execution, pathway-level scalability of the parallel backend, and robustness of rolling-window trajectory analysis under parameter and boundary stress tests.

S11.2 Integrated ES/NES/P-value equivalence. Figure S10 consolidates equivalence evidence across all benchmark regimes and across three output levels: ES, NES, and transformed nominal P-values. Unlike Table S6, which is restricted to the synthetic benchmark summary used in the main-text Figure 1, the inset in Figure S10C recomputes a pooled summary after combining all six validation regimes and is therefore not expected to be numerically identical. Across matched inputs, PyFgsea remained near-identical for NES, machine-precision identical for ES, and statistically faithful for transformed nominal P-values relative to the R reference. The weakest transformed nominal-P concordance occurred in the early trajectory window (Table S7), where only 43 Hallmark pathways passed the size filter and several strongly enriched programs occupied a locally sensitive tail regime. In that setting, moderate absolute nominal-P differences are amplified by the $-\log_{10}(P)$ transform, even though ES remained identical within machine precision and NES stayed near-identical. Accordingly, ES and NES are the more robust primary cross-implementation comparanda in sparse local windows, whereas transformed nominal-P agreement is more sensitive to tail-estimation noise and pathway-count filtering. At the same time, downstream pathway calls remained stable overall: across regimes, the top-10 $|NES|$ pathway overlap ranged from 0.90 to 1.00, and the BH-FDR<0.05 pathway-set overlap ranged from 0.86 to 1.00 (Table S7).

S11.3 Cross-thread determinism under a fixed master seed. Because pathway-level reproducibility under parallel execution is central to the implementation claim, we explicitly audited fixed-seed thread-count invariance before turning to repeated-run stability. Figure S11 shows that pathway-wise outputs and pathway ranking remained invariant across thread counts in this benchmark, directly supporting the deterministic pathway-local PRNG strategy described in Section S9.

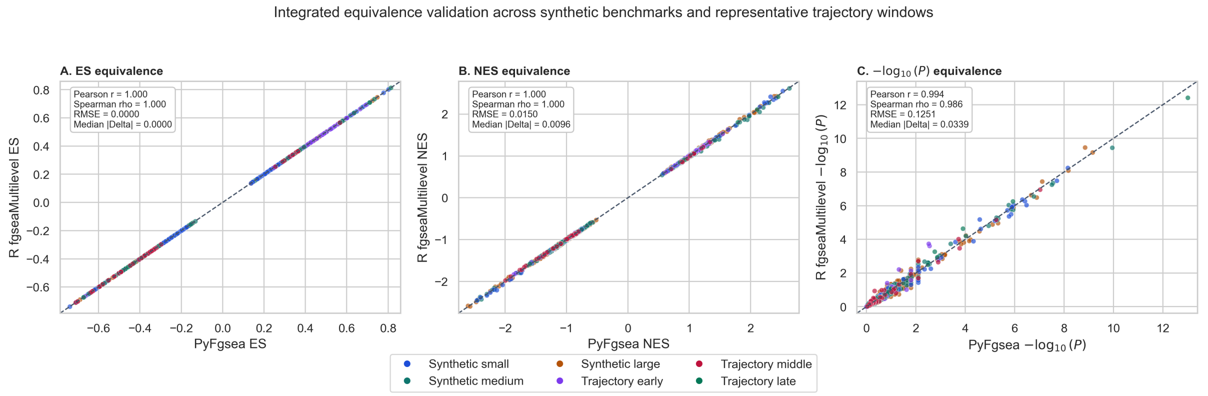

Figure S10: Integrated equivalence validation across synthetic benchmarks and representative trajectory windows. Each panel aggregates pathway-wise outputs from six regimes (three synthetic benchmark sizes plus early, middle, and late erythroid trajectory windows) and compares PyFgsea against the R `fgseaMultilevel` reference for (A) ES, (B) NES, and (C) transformed nominal P-values. Colors indicate benchmark regime; dashed lines indicate identity. The inset in panel C summarizes the pooled six-regime comparison and is therefore not numerically identical to the synthetic-only Figure 1/Table S6 summary reported in the main text.

Table S7: Quantitative equivalence summary across all validation regimes. The expanded table complements the concise main-text metrics by reporting ES, NES, and transformed nominal P-value concordance separately for each synthetic benchmark and each representative erythroid trajectory window; it also summarizes top-10 $|NES|$ pathway overlap and BH-FDR$<$ 0.05 pathway-set overlap between PyFgsea and the R reference within each regime.

Regime	Metric	Pearson r	Spearman ρ	RMSE	Median $\|\Delta\|$	95th pct. $\|\Delta\|$	Top-10 $\|NES\|$ overlap	BH-FDR$<$ 0.05 set overlap
Synthetic large	ES	1.000	1.000	0.0000	0.0000	0.0000	1.000	0.952
Synthetic large	NES	1.000	0.999	0.0145	0.0102	0.0282	1.000	0.952
Synthetic large	Log10P	0.992	0.986	0.1072	0.0301	0.2213	1.000	0.952
Synthetic medium	ES	1.000	1.000	0.0000	0.0000	0.0000	0.900	1.000
Synthetic medium	NES	1.000	0.999	0.0155	0.0095	0.0268	0.900	1.000
Synthetic medium	Log10P	0.997	0.989	0.0941	0.0344	0.2025	0.900	1.000
Synthetic small	ES	1.000	1.000	0.0000	0.0000	0.0000	0.900	0.920
Synthetic small	NES	1.000	1.000	0.0178	0.0072	0.0342	0.900	0.920
Synthetic small	Log10P	0.997	0.994	0.1536	0.0360	0.4010	0.900	0.920
Trajectory early	ES	1.000	1.000	0.0000	0.0000	0.0000	0.900	1.000
Trajectory early	NES	1.000	0.998	0.0135	0.0080	0.0262	0.900	1.000
Trajectory early	Log10P	0.941	0.976	0.3286	0.0693	0.8734	0.900	1.000
Trajectory late	ES	1.000	1.000	0.0000	0.0000	0.0000	0.900	1.000
Trajectory late	NES	1.000	0.997	0.0141	0.0103	0.0255	0.900	1.000
Trajectory late	Log10P	0.996	0.972	0.1880	0.0737	0.4999	0.900	1.000
Trajectory middle	ES	1.000	1.000	0.0000	0.0000	0.0000	0.900	0.857
Trajectory middle	NES	1.000	0.999	0.0163	0.0104	0.0346	0.900	0.857
Trajectory middle	Log10P	0.990	0.940	0.2045	0.0721	0.3980	0.900	0.857

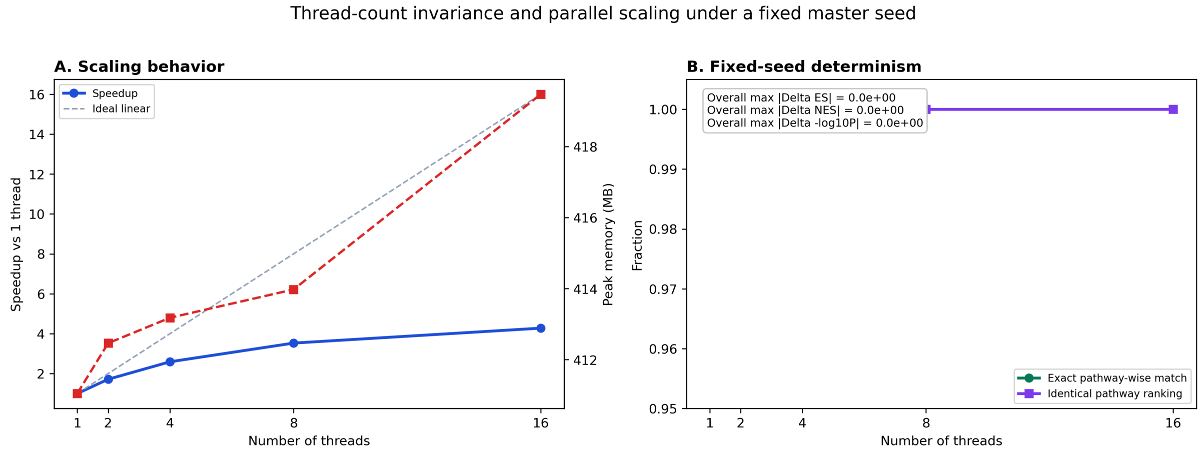

Figure S11: Cross-thread determinism and scaling under a fixed master seed. (A) Pathway-level execution shows near-linear speedup at lower thread counts with only modest peak-memory variation in the enlarged stress-test workload. (B) Deterministic sub-seeding guarantees identical pathway-wise outputs and identical pathway ranking across thread counts in this audit.

S11.4 Fixed-seed reproducibility versus multi-seed variability. Repeated executions should be interpreted in two distinct ways. Under a fixed input and a fixed master seed, the implementation should be deterministic. Under changing seeds, modest variability in multilevel tail estimates is expected because the rare-event estimator remains stochastic. Figure S12 explicitly separates these two regimes. The left panel collapses to nearly degenerate distributions under a fixed seed, whereas the right panel shows the bounded stochastic envelope obtained when varying seeds over repeated runs.

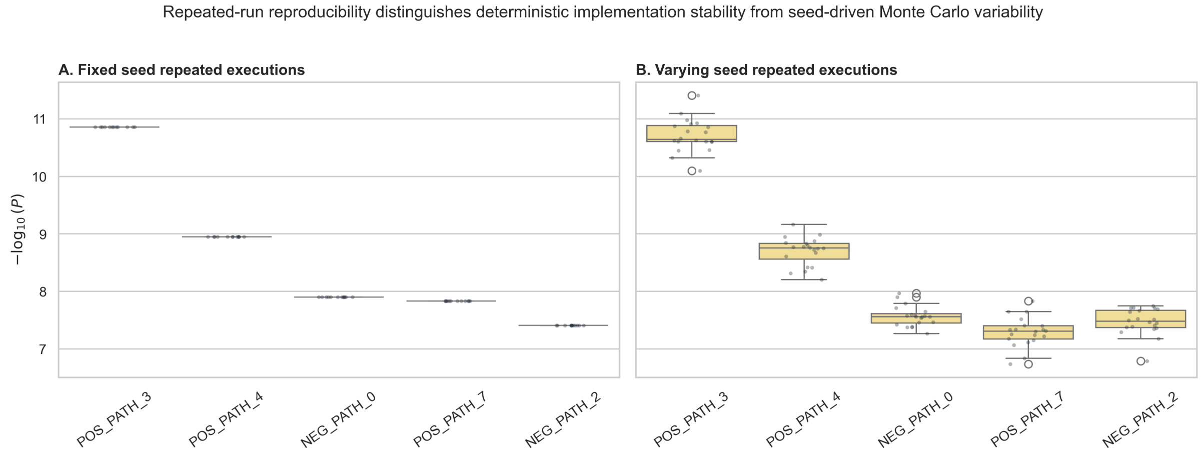

Figure S12: Run-to-run reproducibility audit. Under a fixed seed (left), repeated executions are effectively identical, confirming deterministic end-to-end behaviour. Under varying seeds (right), pathway-level $-\log_{10}(P)$ values fluctuate within a modest stochastic envelope, reflecting the expected Monte Carlo variability of adaptive multilevel sampling rather than implementation instability.

S12. Parallel Execution Validation and Scalability Audit

S12.1 Thread-count invariance under deterministic pathway-local PRNG streams. Section S9 described the design principle: pathway evaluations are conditionally independent

and therefore scheduled in parallel, while each pathway receives a deterministic sub-seed derived from the user-provided master seed and pathway identifier. Figure S11 and Table S8 convert that design claim into an explicit audit. Across thread counts from 1 to 16, pathway-wise outputs remained exactly invariant under a fixed seed, with exact-match and identical-ranking fractions equal to 1.000 throughout this benchmark.

Table S8: Parallel reproducibility audit across thread counts. Exact-match and identical-ranking fractions were computed relative to the 1-thread reference under the same input data and the same master seed. To make peak-memory measurement more reliable than the earlier sampled-RSS proxy, this audit was rerun on an enlarged workload (20,000 genes, 50,000 pathways). For the values reported in Table S8, peak memory was obtained from the Windows subprocess peak working-set statistic. This audit is intended as a within-platform thread-invariance check and is not used for direct cross-table memory comparison with Table S4.

Threads	Time (s)	Speedup	Peak memory (MB)	Exact match	Rank match	Max $\lvert \Delta NES \rvert$	Max $\lvert \Delta - \log_{10} P \rvert$
1	5.502	1.00	411.0	1.000	1.000	0.0e+00	0.0e+00
2	3.209	1.71	412.5	1.000	1.000	0.0e+00	0.0e+00
4	2.123	2.59	413.2	1.000	1.000	0.0e+00	0.0e+00
8	1.559	3.53	414.0	1.000	1.000	0.0e+00	0.0e+00
16	1.286	4.28	419.5	1.000	1.000	0.0e+00	0.0e+00

S12.2 Stateful versus stateless rolling-window execution. To isolate the practical benefit of the stateful runner used in trajectory analysis, we benchmarked repeated trajectory-derived score vectors against a 3000-pathway stress-test collection. As shown in Figure S15, reusing pathway definitions and NES background structures yielded a consistent end-to-end wall-time advantage over repeatedly calling stateless preranked GSEA. The gain was stable across 100, 300, and 1000 windows (roughly 1.9-fold in this conservative end-to-end stress test), demonstrating that the rolling-window runner reduces repeated overhead without changing the per-window statistical target. This value should be interpreted alongside the larger 7.47-fold speedup reported in the original Supplementary Table S3: that earlier number came from a narrower 100-window component benchmark with 5000 pathways and therefore emphasized amortized preprocessing and NES-background reuse more strongly. We therefore report the conservative end-to-end figure first and interpret the larger number as a scope-specific component benchmark rather than a universal whole-workflow gain.

S13. Rolling-Window Robustness and Parameter Stress Tests

S13.1 Window-size and step-size stress test. The original sensitivity addition in Figure S8 already demonstrated that both window size and step size influence the shape of NES trajectories for three representative pathways. Here we summarize the same grid more explicitly in a reviewer-oriented form. Figure S13 converts the full 3×3 window-by-step grid into compact summaries of smoothness, peak-position drift, significant-window overlap, and runtime. Smaller windows increased temporal responsiveness but amplified stepwise NES roughness and reduced agreement with the baseline setting; coarse step sizes reduced runtime but also reduced significant-window overlap by undersampling local transients.

S13.2 Boundary-handling audit at trajectory termini. Reviewer 2 also asked for clearer boundary handling. To make the edge behaviour more transparent, Figure S14 now separates the audit into three complementary views: the changing cell count of terminal windows, representative pathway trajectories, and a pathway-level continuity summary relative to the last full window. The main-text Figure 2 uses the default full-window workflow only, whereas the audit here evaluates a separate terminal-truncation extension that adds progressively smaller tail

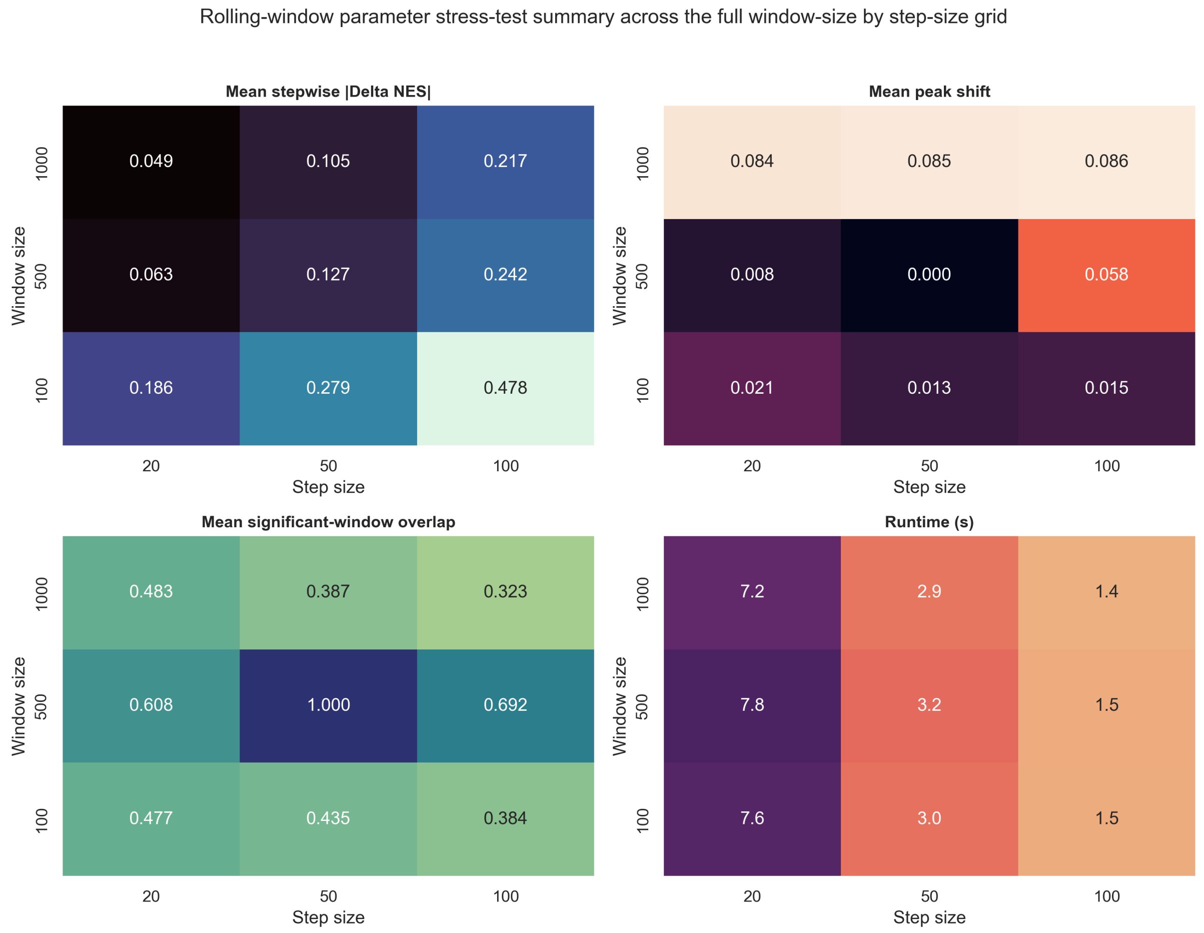

Figure S13: Rolling-window parameter stress-test summary over the full window-size by step-size grid. Heatmaps summarize four practical readouts averaged across three representative pathways: stepwise NES roughness, peak-position shift relative to the baseline setting (window 500, step 50), significant-window overlap, and runtime.

Table S9: Rolling-window parameter sensitivity summary across the full grid. The table reports the effective window/step percentages relative to the erythroid trajectory, the number of windows, average smoothness and peak-shift diagnostics, significant-window overlap relative to the baseline setting (window 500, step 50), and measured runtime.

Window	Step	Window (%)	Step (%)	Windows	Median stepwise $\|\Delta NES\|$	Peak-shift	Sig. overlap	Runtime (s)
100	20	2.8	0.6	174	0.1858	0.0210	0.477	7.6
100	50	2.8	1.4	70	0.2792	0.0130	0.435	3.0
100	100	2.8	2.8	35	0.4780	0.0152	0.384	1.5
500	20	14.0	0.6	154	0.0627	0.0083	0.608	7.8
500	50	14.0	1.4	62	0.1273	0.0000	1.000	3.2
500	100	14.0	2.8	31	0.2423	0.0576	0.692	1.5
1000	20	28.0	0.6	129	0.0490	0.0841	0.483	7.2
1000	50	28.0	1.4	52	0.1054	0.0854	0.387	2.9
1000	100	28.0	2.8	26	0.2166	0.0857	0.323	1.4

windows without any padding. The resulting NES trajectories continue smoothly rather than showing abrupt edge artifacts. Quantitatively, the truncation-only tail windows retain high pathway-level agreement with the last full window, supporting the use of non-padded terminal handling while making the end-of-trajectory behaviour visually explicit.

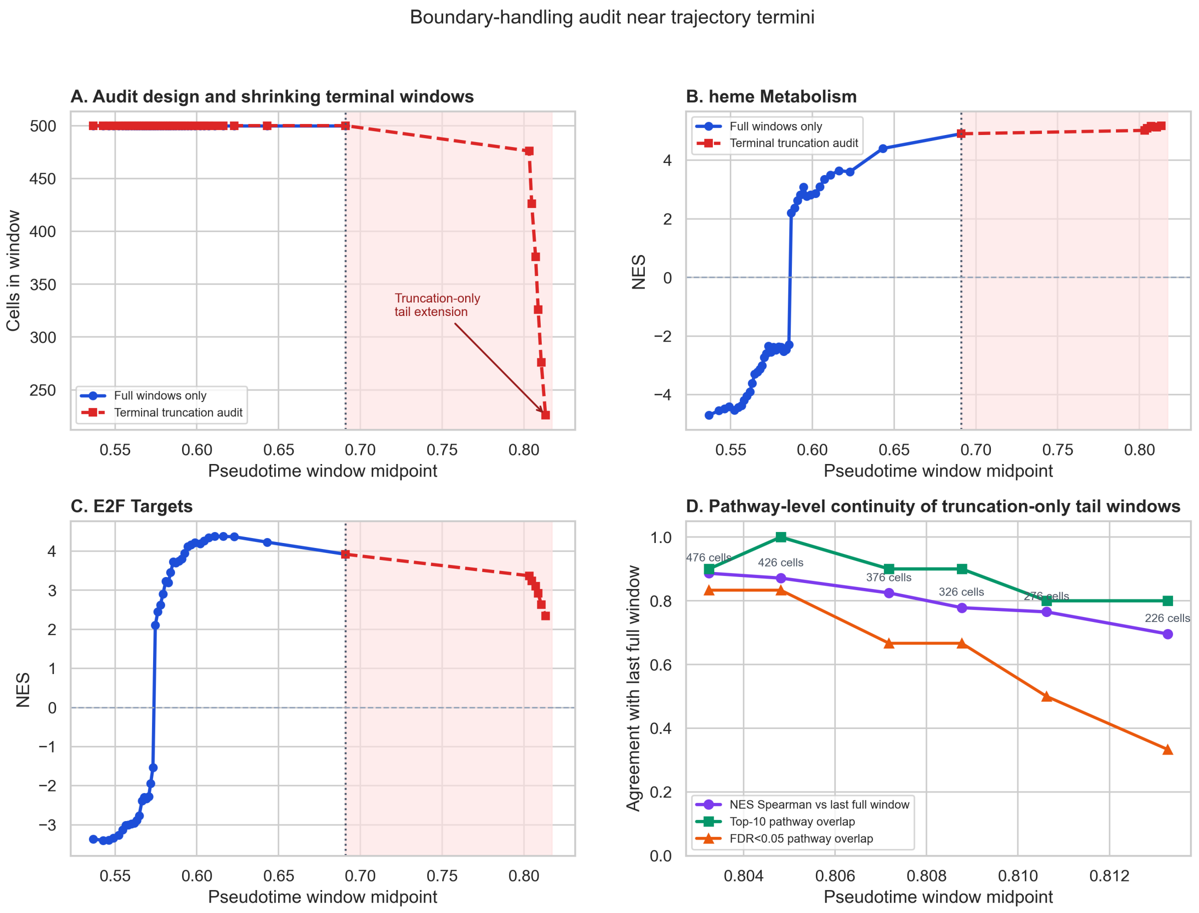

Figure S14: Boundary-handling audit near the trajectory terminus. (A) Audit design: terminal truncation progressively shortens tail windows beyond the last full 500-cell window instead of padding the trajectory end. (B–C) Representative pathway trajectories showing that the truncation extension continues the local NES trend smoothly for heme metabolism and E2F targets. (D) Quantitative continuity summary for truncation-only tail windows relative to the last full window, reported as pathway-level Spearman correlation of NES values and top-10 pathway overlap.

S13.3 Practical parameter guidance. Taken together, Figures S8, S13, and S14 support the empirical guidance used in the revised main text. For exploratory scans, window sizes near 5–10% of cells with step sizes near 1–2% of cells provide a practical starting range, but the setting should still be tuned to trajectory length, local noise, and the desired degree of smoothing. The current erythroid grid brackets rather than directly centers this generic range. The 500-cell baseline used for the erythroid illustration is deliberately somewhat larger than this starting range because that lineage segment is comparatively short and noisy; in practice, larger windows are appropriate when the goal is a smoother descriptive profile rather than fine transition localization. Smaller windows should be interpreted as high-resolution but noise-sensitive views, whereas larger windows behave more like smoothed summaries of broad trajectory trends. Section S15.4 below additionally tests this guidance in an external non-erythroid raw-count trajectory with stronger dropout structure and again finds that the 8% window / 1–2% step region

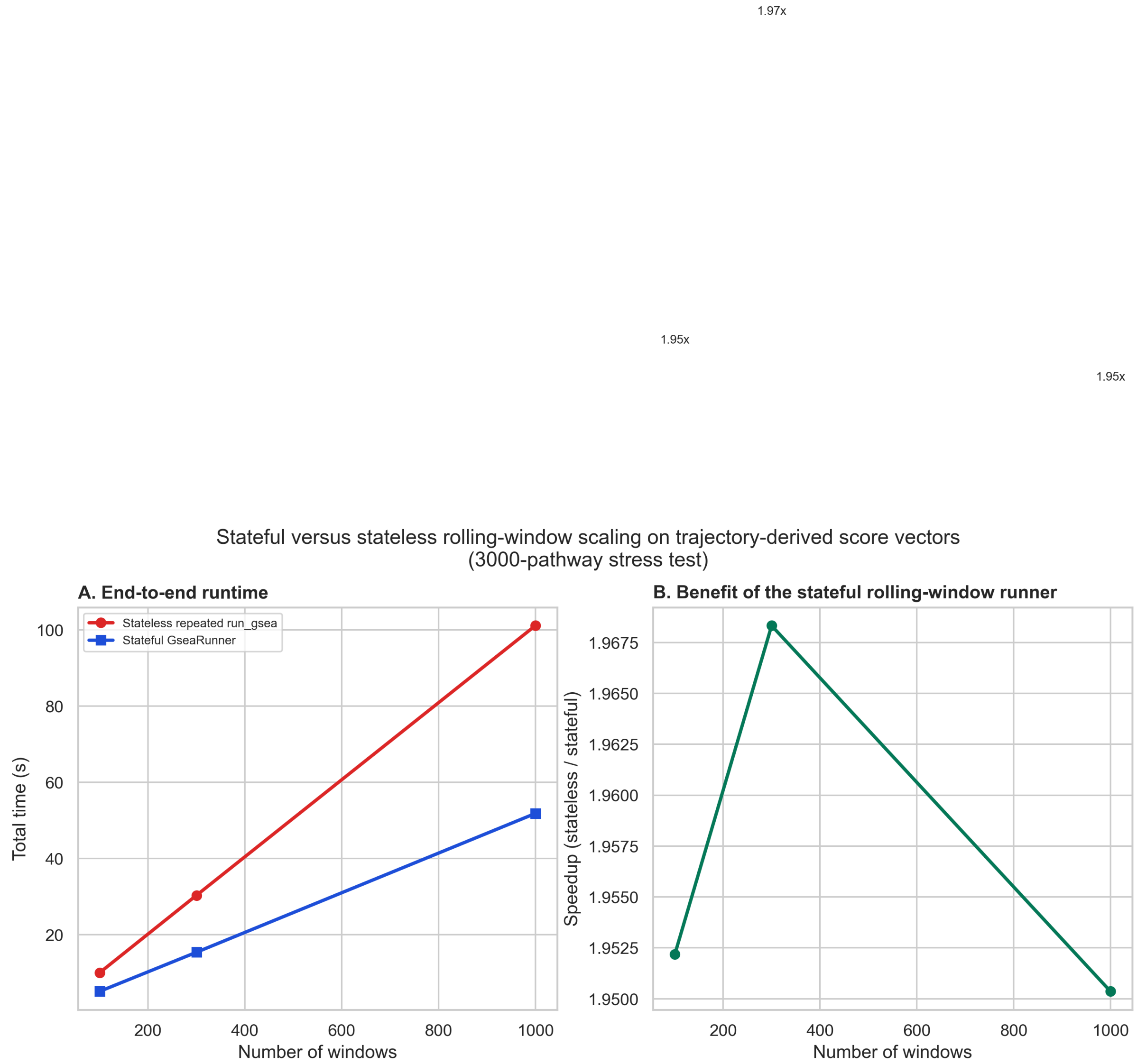

Figure S15: Stateful versus stateless rolling-window scaling on trajectory-derived score vectors under a 3000-pathway stress test. Reusing cached pathway definitions and NES background structures yields a consistent end-to-end wall-time advantage as the number of windows increases.

provides the best overall balance across stability diagnostics.

S14. Summary of Validated Claims and Practical Guidance

The revised supplement now closes the loop between algorithm description and observable software behaviour. Table S10 maps each major manuscript claim to the concrete experiment, metric, and empirical conclusion supporting it.

S15. Limitation Audit of the Rolling-Window Ranking Statistic

The revised Discussion now explicitly acknowledges two practical limitations of the rolling-window ranking statistic used in trajectory analysis: (i) genes or pathways with complex non-monotonic dynamics may be underestimated, increasing false-negative risk; and (ii) sparse, high-variance genes may be over-amplified, creating local false-positive peaks. To make these caveats directly observable rather than merely verbal, we added the targeted audit below. The goal is not to defend the statistic as universally optimal, but to show where it behaves stably, where it begins to fail, and how simple sensitivity checks can help users interpret local pathway peaks more cautiously.

S15.1 Real-data examples of how the limitation manifests. Figure S16 uses four representative genes from the real erythroid pseudotime analysis to visualize the relationship between smoothed single-gene expression, the actual rolling-window ranking signal used by the method, and a related Hallmark pathway NES trajectory. Monotonic programs (for example, HBB or IFI27) produce ranking traces that remain easy to interpret. In contrast, transient or non-monotonic genes (for example, MKI67 and JUNB) generate ranking traces that are broader, more asymmetric, or more locally mixed than the underlying smoothed expression profile, illustrating why temporally localized biology can be blurred or phase-shifted by the window-versus-rest statistic. Because the released erythroid matrix is a dense standardized layer rather than a raw-count matrix with preserved dropout structure, the sparse/high-variance failure mode is audited below through semi-simulation rather than by raw-gene plots alone.

S15.2 Semi-simulated audit of false-negative and false-positive regimes. To test these limitations more directly, we embedded synthetic pathway genes into the real erythroid pseudotime cell ordering while keeping a large background of real standardized genes. Four pathway archetypes were examined: a coherent monotonic program, a narrow transient pulse, a mixed biphasic program consisting of early- and late-peaking subgroups, and a sparse high-variance burst process with no latent biological trajectory. Figure S17 overlays the latent pathway signal with the observed NES trajectories returned by the current rolling-window statistic. The monotonic pathway remained the most stable reference case. The transient pathway remained detectable but its peak shifted slightly earlier than the latent optimum. The mixed biphasic pathway showed a markedly smaller number of significant windows despite matched per-gene amplitude, because early and late subprograms diluted each other within the same pathway. Finally, the sparse-noise pathway had a flat latent truth yet still produced localized positive NES peaks, directly illustrating the false-positive mechanism discussed in the revised main-text Discussion.

S15.3 Sensitivity-check control for sparse/high-variance pathways. We did not replace the main ranking statistic in the revised manuscript. However, as a sensitivity check, Figure S18 compares the current trajectory workflow against a simple detection-rate-weighted

Table S10: Validated-claims summary linking manuscript claims to the new supplementary validation package.

Manuscript claim	Validation experiment	Metric(s)	Result and reference
PyFgsea remains statistically aligned with `fgseaMultilevel`	Integrated cross-regime equivalence audit	ES/NES/$-\log_{10}(P)$ concordance	NES remained uniformly near-identical; ES was identical within machine precision; transformed nominal P-values were statistically faithful (Figure S10, Tables S6 and S7)
Parallel execution is reproducible under a fixed master seed	Cross-thread determinism audit from 1 to 16 threads	Exact-match fraction, identical-ranking fraction, max absolute differences	All audited thread counts yielded exact pathway-wise matches and identical pathway ranking in this benchmark (Figure S11, Table S8)
Repeated runs separate implementation stability from expected Monte Carlo variability	Fixed-seed and varying-seed repeated executions	Distribution of pathway-wise $-\log_{10}(P)$ over repeated runs	Fixed-seed reruns collapsed to effectively identical outputs, whereas changing seeds produced only bounded stochastic tail variation (Figure S12)
Rolling-window conclusions are robust but parameter-dependent	Multi-pathway window/step stress test plus terminal boundary audit	NES roughness, peak shift, significant-window overlap, runtime	Smaller windows and coarser steps trade stability for resolution and runtime; baseline settings preserved the best overlap while terminal handling remained smooth and non-padded (Figures S8, S13, and S14; Table S9)
Parameter guidance and peak-diagnostic workflow generalize beyond the erythroid illustration	External raw-dropout stress test on GSE126085	3×3 window/step stability summary, detection-rate drift, weighted-check attenuation	The 8% window / 1–2% step region again remained the most balanced; a stable glycolysis program was preserved while a low-detection UV response up peak was attenuated (Figure S19)
The stateful trajectory runner reduces repeated computational overhead	Stateful-versus-stateless trajectory stress test	End-to-end wall-time speedup	A conservative 3000-pathway end-to-end stress test showed an approximately 1.9-fold wall-time advantage, consistent with the larger 7.47-fold gain reported in the narrower 100-window component benchmark of the original Supplementary Table S3 (Figure S15)

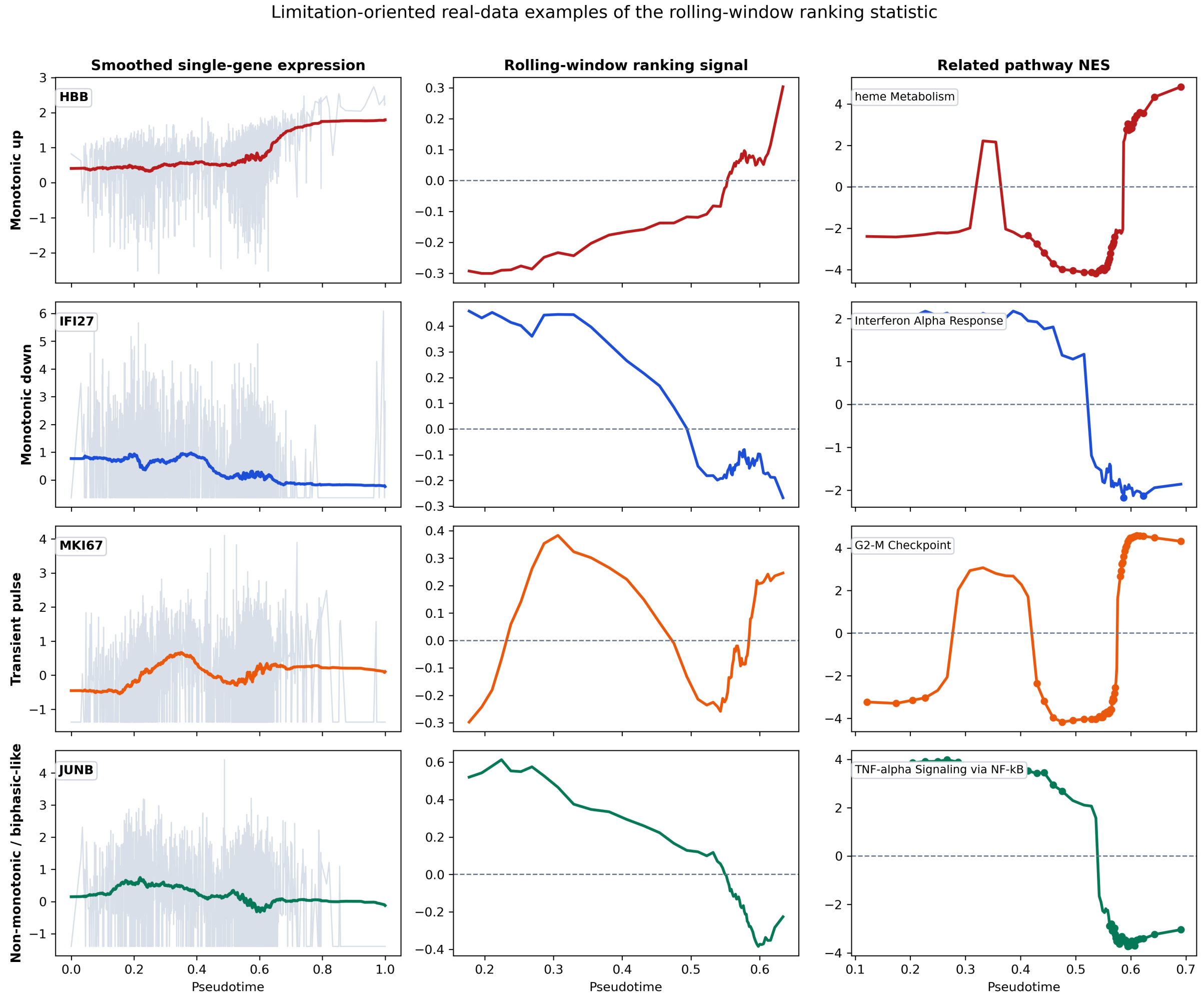

Figure S16: Real-data limitation-oriented examples from the erythroid trajectory. Each row shows (left) the smoothed single-gene expression profile, (middle) the corresponding rolling-window ranking signal used by the method, and (right) a related Hallmark pathway NES curve under the same rolling-window settings. Monotonic genes produce comparatively stable ranking traces, whereas transient or non-monotonic genes generate broader or locally mixed ranking signals, illustrating the mechanism underlying potential false-negative pathway calls for complex temporal programs.

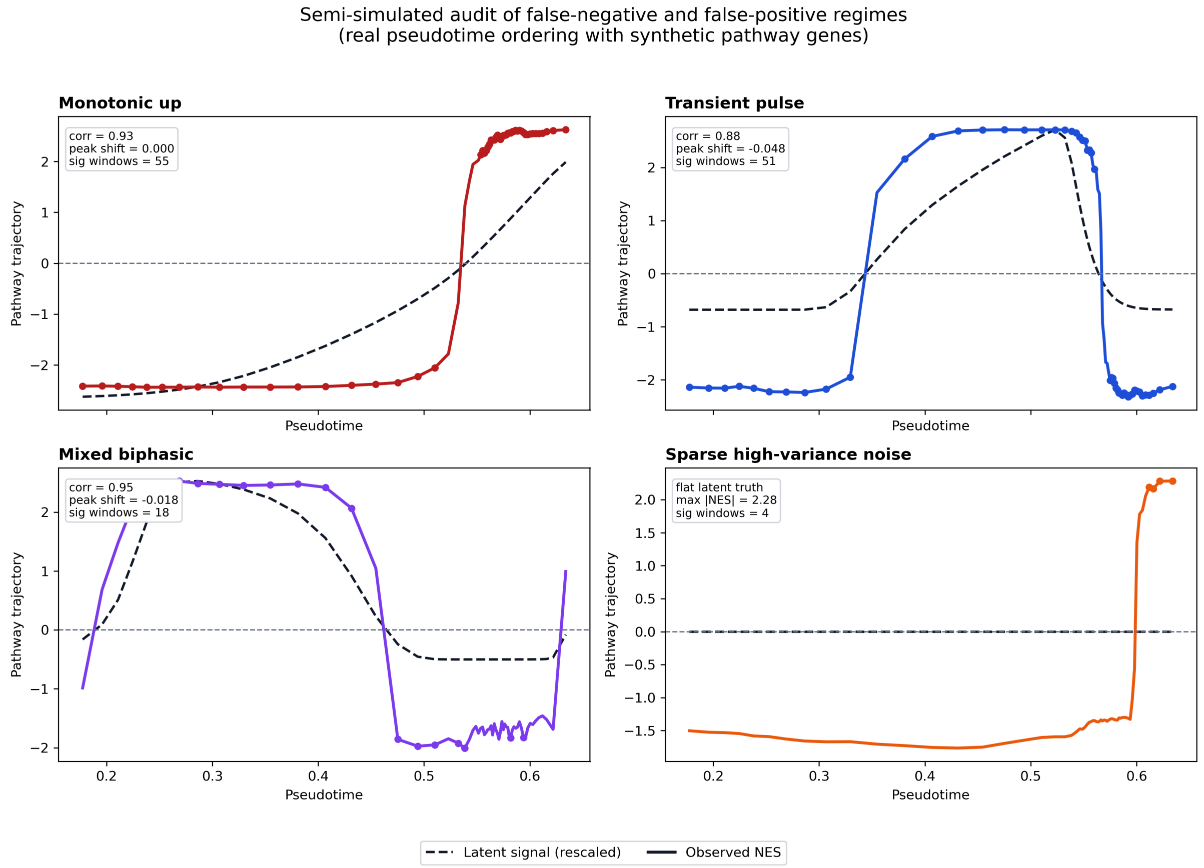

Figure S17: Semi-simulated limitation audit on the real erythroid pseudotime ordering. Dashed curves show the latent pathway signal (rescaled for visual comparison), whereas colored curves show the observed NES trajectories returned by the current rolling-window statistic. The monotonic pathway behaves stably; the narrow transient pulse is shifted slightly; the mixed biphasic pathway is attenuated and yields fewer significant windows; and the sparse high-variance pathway shows local positive NES peaks despite a flat latent truth.

control that downweights genes observed in only a tiny fraction of cells. This control is not proposed as the new default method; it is included only to test whether the sparse/high-variance failure mode is real. The result is informative: monotonic and transient pathways change very little, whereas the sparse-noise pathway loses its false-positive windows under the weighted control. This reinforces the interpretation that extreme local peaks should be cross-checked when they are driven by very low-detection genes.

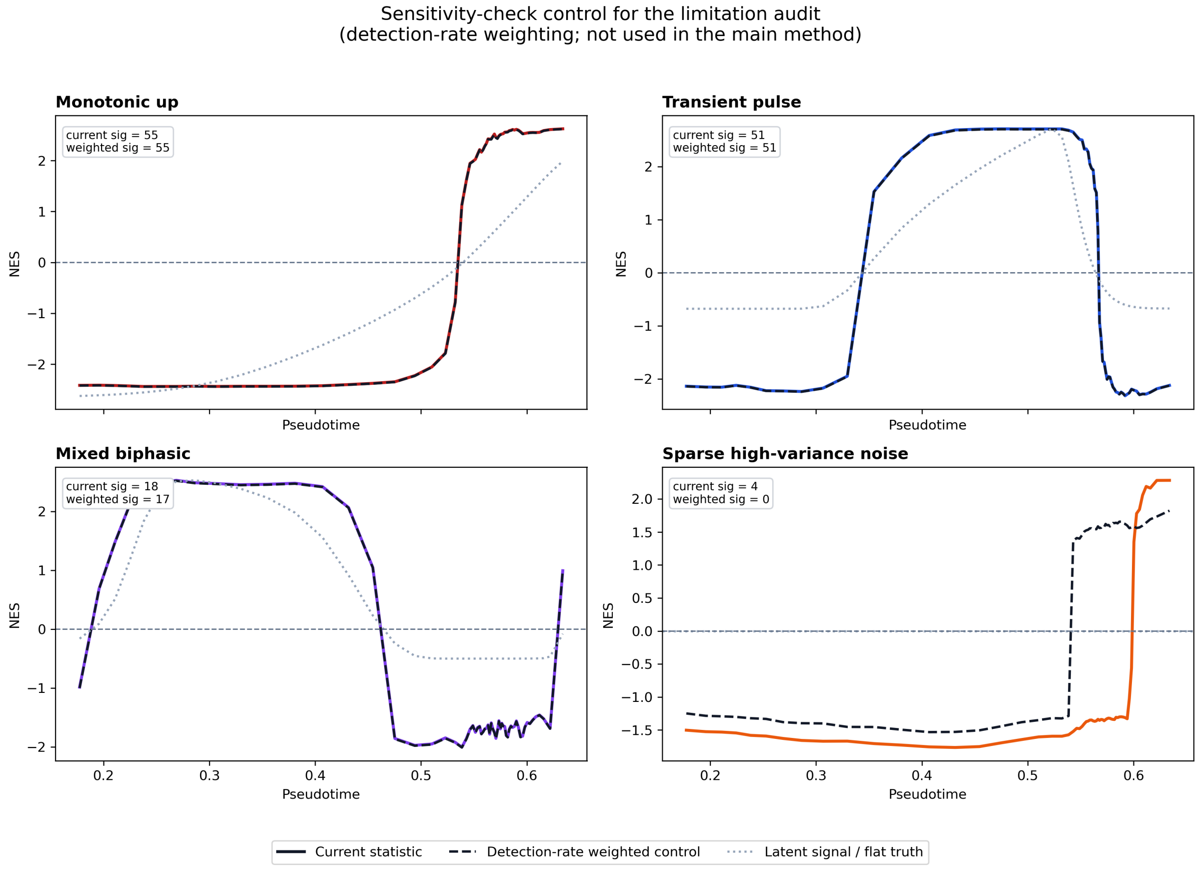

Figure S18: Sensitivity-check control for the limitation audit. Solid lines show the current rolling-window statistic, dashed black lines show a simple detection-rate-weighted control, and dotted gray lines show the latent pathway signal or flat latent truth. The control barely changes the monotonic baseline but attenuates the sparse-noise pathway enough to remove its significant windows, supporting the interpretation that low-detection, high-variance genes can drive local false-positive enrichments.

S15.4 External raw-dropout stress test on GSE126085. GSE126085 is a human CD14+ monocyte-to-macrophage M-CSF time-series and therefore provides a biologically relevant non-erythroid trajectory context. However, because the raw-count data exhibit pronounced detection-rate drift and only partially monotonic temporal ordering, we use this dataset here as an external raw-dropout stress test rather than as a biological showcase. Figure S19 summarizes four practical observations. First, this real raw-count trajectory is substantially harsher than the erythroid illustration, with median detected genes drifting by orders of magnitude across rolling windows. Second, a 3×3 window-by-step grid centered directly on the main-text starting range again supports window sizes near 8% and step sizes near 1–2% as the most balanced region; coarser 4% steps reduced overlap and stability, with significant-window overlap falling from 1.00 at the 8%/2% reference to 0.58 at 8%/4% and 0.38 at 12%/4%. Third, a stable program (glycolysis) remained largely preserved under the detection-rate-weighted sensitivity check. Fourth, a sus-

picious local peak (UV response up) occurred in an extremely low-detection window and was strongly attenuated by the same diagnostic weighting. These pathway examples are included as methodological exemplars rather than as prioritized pathway-specific biological claims. We therefore interpret this analysis as external support that the practical parameter guidance is not erythroid-specific and that detection-linked local peaks can be diagnosed in real raw-count data without redefining the primary statistic.

Table S11: Compact summary of the limitation-oriented audit. Corr. denotes the correlation between the latent pathway signal and the observed NES trajectory in the semi-simulation; peak shift is reported in pseudotime units. The detection-rate-weighted control is included as a sensitivity check only and is not the primary method used in the main manuscript.

Pattern	Corr. (current)	Peak shift	Sig. windows	Weighted sig.	Max $\lvert NES \rvert$ (cur→wt)	Interpretation
Monotonic up	0.93	0.000	55	55	2.63→2.62	Monotonic pathway behaviour remained stable and served as the baseline reference.
Transient pulse	0.88	-0.048	51	51	2.71→2.70	The narrow pulse remained detectable but its peak shifted slightly earlier than the latent optimum.
Mixed biphasic	0.95	-0.018	18	17	2.53→2.52	Early and late subprograms diluted each other, reducing the number of significant windows despite matched per-gene amplitude.
Sparse high-variance noise	–	–	4	0	2.28→1.82	Sparse high-variance bursts generated local false-positive windows; a simple detection-rate-weighted control attenuated them.

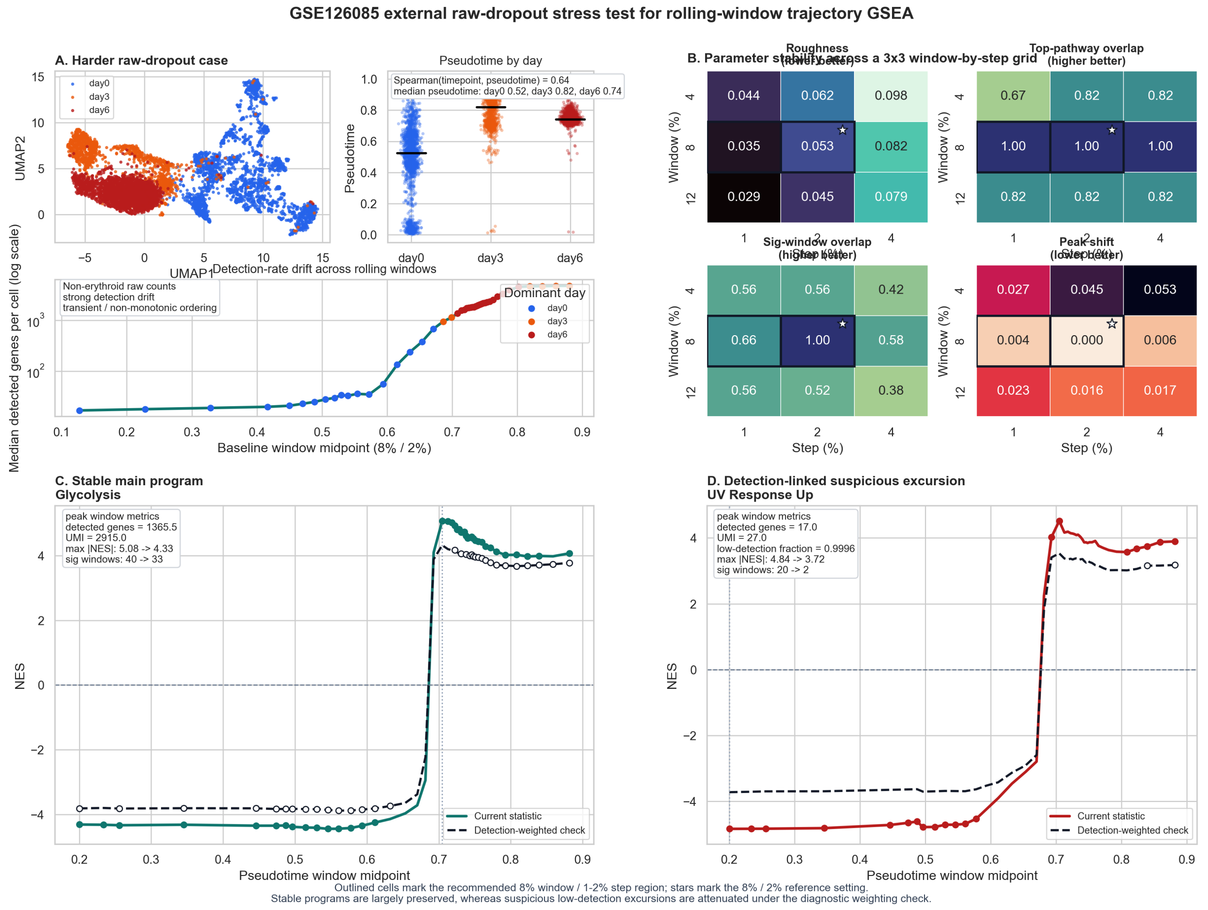

Figure S19: External raw-dropout stress test of rolling-window trajectory GSEA on GSE126085. (A) A non-erythroid raw-count single-cell time-series with strong detection-rate drift and imperfectly monotonic temporal ordering, used here as a harder external stress-test rather than a biological showcase. The upper panels show UMAP grouping and pseudotime distributions across day0/day3/day6; the lower panel shows the pronounced drift in median detected genes across rolling windows under the reference setting (8% window, 2% step). (B) Parameter stability summary over a 3×3 grid centered on the main-text practical starting range. Window sizes near 8% with step sizes near 1–2% provided the best overall balance between smoothness, pathway overlap, significant-window overlap, and peak stability, whereas coarser 4% steps reduced stability. Outlined cells denote the recommended 8% window / 1–2% step region, and stars mark the 8% / 2% reference setting. (C) Example of a stable main program (glycolysis): the detection-weighted diagnostic check only modestly attenuates the trajectory profile, preserving the main signal. (D) Example of a detection-linked suspicious excursion (UV response up): the peak occurs in an extremely low-detection window and is strongly attenuated by the diagnostic weighting check, consistent with a local low-detection artefact rather than a comparably stable pathway program. Panels C and D are methodological exemplars rather than prioritized pathway-specific biological claims.
